# Childhood severe acute malnutrition is associated with metabolomic changes in adulthood

**DOI:** 10.1172/jci.insight.141316

**Published:** 2020-11-17

**Authors:** Debbie S. Thompson, Celine Bourdon, Paraskevi Massara, Michael S. Boyne, Terrence E. Forrester, Gerard Bryan Gonzales, Robert H. J. Bandsma

**Affiliations:** 1 Translational Medicine Program, Hospital for Sick Children, Toronto, Canada.; 2 Centre for Global Child Health, Hospital for Sick Children, Toronto, Canada.; 3 Caribbean Institute for Health Research, The University of the West Indies, Kingston, Jamaica.; 4 The Childhood Acute Illness & Nutrition Network, Nairobi, Kenya.; 5 Department of Nutritional Sciences, Faculty of Medicine, University of Toronto, Toronto, Canada.; 6 Department of Medicine, The University of the West Indies, Kingston, Jamaica.; 7 University of the West Indies Solutions for Developing Countries, Kingston, Jamaica.; 8 Gastroenterology, Department of Internal Medicine and Pediatrics, Ghent University, Ghent, Belgium.; 9 Nutrition, Metabolism and Genomics Group, Division of Human Nutrition and Health, Wageningen University, Wageningen, the Netherlands.; 10 Division of Gastroenterology, Hepatology and Nutrition, Hospital for Sick Children, Toronto, Canada.

**Keywords:** Metabolism, Cardiovascular disease, Diabetes

## Abstract

**BACKGROUND:**

Severe acute malnutrition (SAM) is a major contributor to global mortality in children under 5 years. Mortality has decreased; however, the long-term cardiometabolic consequences of SAM and its subtypes, severe wasting (SW) and edematous malnutrition (EM), are not well understood. We evaluated the metabolic profiles of adult SAM survivors using targeted metabolomic analyses.

**METHODS:**

This cohort study of 122 adult SAM survivors (SW = 69, EM = 53) and 90 age-, sex-, and BMI-matched community participants (CPs) quantified serum metabolites using direct flow injection mass spectrometry combined with reverse-phase liquid chromatography. Univariate and sparse partial least square discriminant analyses (sPLS-DAs) assessed differences in metabolic profiles and identified the most discriminative metabolites.

**RESULTS:**

Seventy-seven metabolite variables were significant in distinguishing between SAM survivors (28.4 ± 8.8 years, 24.0 ± 6.1 kg/m^2^) and CPs (28.4 ± 8.9 years, 23.3 ± 4.4 kg/m^2^) (mean ± SDs) in univariate and sPLS-DA models. Compared with CPs, SAM survivors had less liver fat; higher branched-chain amino acids (BCAAs), urea cycle metabolites, and kynurenine/tryptophan (KT) ratio (*P* < 0.001); and lower β-hydroxybutyric acid and acylcarnitine/free carnitine ratio (*P* < 0.001), which were both associated with hepatic steatosis (*P* < 0.001). SW and EM survivors had similar metabolic profiles as did stunted and nonstunted SAM survivors.

**CONCLUSION:**

Adult SAM survivors have distinct metabolic profiles that suggest reduced β-oxidation and greater risk of type 2 diabetes (BCAAs, KT ratio, urea cycle metabolites) compared with CPs. This indicates that early childhood SAM exposure has long-term metabolic consequences that may worsen with age and require targeted clinical management.

**FUNDING:**

Health Research Council of New Zealand, Caribbean Public Health Agency, Centre for Global Child Health at the Hospital for Sick Children. DST is an Academic Fellow and a Restracomp Fellow at the Centre for Global Child Health. GBG is a postdoctoral fellow of the Research Foundation Flanders.

## Introduction

Worldwide, an estimated 16.6 million children under the age of 5 years are severely wasted (1), and severe acute malnutrition (SAM) remains a significant contributor to global mortality (1). As more children survive episodes of SAM, there is a growing need to understand whether this early life exposure is associated with long-term health risks, including the development of noncommunicable diseases (NCDs). Unlike prenatal undernutrition, which has been associated with increased rates of type 2 diabetes (T2D), hypertension, coronary heart disease, and stroke in adulthood (2–4), the long-term consequences of SAM in early childhood are poorly understood.

Limited data link early childhood growth failure to increased cardiovascular disease risk (dyslipidemia, hypertension, and glucose intolerance) in later life (5). Norwegian children with below-average weight and BMI in early life experienced more cardiovascular events by 64 years of age, but only after undergoing rapid weight rebound in later childhood (6). Childhood exposure to the Chinese famine (from 1959 through 1962) was also shown to predict increased diabetes risk in adulthood, specifically among those who experienced the most severe famine conditions (7). Similarly, adults exposed to the Biafran famine in early childhood had an increased prevalence of high blood pressure (8); however, the study lacked birth weight data and was therefore unable to separate the effects of fetal and infant famine exposure (8).

Acutely, SAM (defined by weight-for-height *z* score < –3, mid-upper arm circumference < 115 mm, and/or bilateral pitting edema) (9) is associated with several alterations in intermediary metabolism, normally tightly regulated by hormones such as insulin and glucagon. Insulin secretion is impaired during SAM (10, 11) and was shown to remain impaired up to 6 weeks after the body weight of children improved (12). While Malawian children diagnosed with SAM had profoundly different metabolic profiles from controls after hospital discharge (13), we recently reported that 7 years posttreatment, their metabolic profiles were similar (14). Additionally, the 2 clinical phenotypes of SAM (severe wasting and edematous malnutrition) have different intermediary metabolism during the acute illness, as illustrated by differences in endogenous glucose production (15), lipolysis, and lipid oxidation (16). These differences have been reported to resolve with nutritional recovery (16). However, knowing that NCDs develop later in life, often in the context of chronic obesity, conclusions about persisting metabolic effects of SAM are difficult to draw based on studies in these younger cohorts.

There is a paucity of data regarding the metabolic profiles of adult SAM survivors. Early postnatal malnutrition was associated with decreased insulin sensitivity and glucose intolerance in young Mexican men, independent of birth weight (17). Conversely, Jamaican adult SAM survivors and controls were reported to have similar insulin sensitivity, insulin clearance, and adiponectin levels (18), but survivors of severe wasting had decreased glucose tolerance and worse pancreatic β cell function than survivors of edematous malnutrition (19). The 2 SAM phenotypes also showed differential patterns of gene methylation as adults, particularly in immune, metabolic, body composition, and cardiovascular pathways (20), but a control group was not available for comparison. We therefore conducted metabolomic analyses in a subset of these individuals, because while the evidence suggests greater cardiometabolic risk in some adult SAM survivors, both the mechanisms and the metabolic intermediaries remain poorly understood.

This study is the first to our knowledge to compare the metabolic profiles of adult SAM survivors with those of matched community participants and relate these profiles to the risk of T2D, hypertension, and fatty liver disease. Survivors of severe wasting and edematous malnutrition were also contrasted to understand if their metabolic presentations are divergent in adulthood. We hypothesized that adult SAM survivors, especially those who experienced severe wasting, would present with a metabolic profile that suggests greater cardiometabolic risk compared with unexposed controls.

## Results

### Participant characteristics.

The clinical characteristics of the adult SAM survivors (*n* = 69 survivors of severe wasting; *n* = 53 survivors of edematous malnutrition) and 90 community participants selected for metabolomic analysis are described in Table 1. This subset of 122 adult SAM survivors were similar (with respect to age, sex, and BMI) to the other 194 participants with whom they were enrolled and the 297 subjects who could have been available for enrollment. SAM survivors and community participants had similar socioeconomic status, and in line with findings from the full cohort, this subset of SAM survivors had shorter stature (*P* = 0.03) and higher L/S ratio, i.e., less liver fat (*P* < 0.01), compared with community participants. Furthermore, survivors of severe wasting weighed less (*P* < 0.001) and had lower BMI (*P* < 0.001), waist circumference (*P* < 0.01), fat mass (*P* < 0.01), android fat (*P* = 0.013), and lean mass (*P* = 0.04) than survivors of edematous malnutrition. Also, in this subset, survivors of severe wasting still tended toward worse pancreatic β cell function (IGI) compared with survivors of edematous malnutrition (*P* = 0.052).

Clinical differences between survivors of SAM and community participants were most apparent in men. A sex-stratified analysis showed that male survivors of SAM were shorter (170.6 ± 7.8 cm vs. 176.0 ± 7.1 cm; *P* < 0.001), weighed less (63.6 ± 12.5 kg vs. 68.1 ± 9.4 kg; *P* = 0.03), and had lower lean mass (53.5 ± 7.8 kg vs. 57.5 ± 6.6 kg; *P* = 0.003) but had higher android-to-gynoid fat (AG) ratio (0.92 ± 0.19 vs. 0.85 ± 0.19; *P* = 0.047) and L/S ratio, i.e., less liver fat (1.23 ± 0.1 vs. 1.17 ± 0.1; *P* = 0.034), than male community participants (Supplemental Table 1; supplemental material available online with this article; https://doi.org/10.1172/jci.insight.141316DS1). Furthermore, this “small” phenotype among male SAM survivors was mainly driven by those who had experienced severe wasting because they were lighter (60.3 ± 11 kg vs. 69.1 ± 14 kg; *P* = 0.005), with a lower BMI (20.6 [19.2–22.5] kg/m^2^ vs. 22.9 [20–24.8] kg/m^2^; *P* = 0.02) and lean mass (51.5 ± 7.6 kg vs. 56.8 ± 7 kg; *P* = 0.007), than survivors of edematous malnutrition (data not shown). Similar to males, the “small” phenotype was also observed in women who survived severe wasting because they had lower weight (64.1 ± 18 kg vs. 75.2 ± 19 kg; *P* = 0.027), lower BMI (23.3 [20.2–27.6] kg/m^2^ vs. 29.8 [21.6–32.5] kg/m^2^; *P* = 0.026), smaller waist circumference (76.3 [70.4–88.8] cm vs. 90.4 [76.2–101] cm; *P* = 0.049), and less lean mass (38.1 ± 5.7 kg vs. 41.6 ± 5.5 kg; *P* = 0.02) than women who survived edematous malnutrition. Women who survived severe wasting also had a lower oDI (186, range 118–232, vs. 279, range 197–368; *P* = 0.035) and a tendency toward lower mean liver attenuation (*P* = 0.07) than women who survived edematous malnutrition (data not shown).

### Metabolite detection and quantification.

Of the 143 metabolites screened by the University of Alberta’s The Metabolomics Innovation Centre (TMIC) PRIME Assay and direct injection mass spectrometry (DIMS) with a reverse-phase liquid chromatography and tandem mass spectrometry (LC-MS/MS) system, 130 metabolites passed the predetermined quality control cutoffs, including 13 biogenic amines, 22 amino acids, 14 lysophosphatidylcholines, 10 sphingomyelins and 10 phosphatidylcholines, 40 acylcarnitines, 16 organic acids, 1 monosaccharide, and 4 other molecules (Supplemental Table 2, P = 0.077; for β-hydroxybutyric acid model, *P* = 0.052). Supplemental Table 1 presents the median concentrations of all measured metabolites, 7 calculated summary variables (e.g., total, essential, aromatic [AAA], branched-chain [BCAA], glucogenic, and ketogenic amino acids), and 13 ratio variables, such as the kynurenine/tryptophan (KT) ratio and the Fischer ratio. Only 2 values were below the detection range and were replaced by half the limit of detection (LOD) of that metabolite (hippuric acid). No sample outlier or inherent clustering was detected by principal component analysis (PCA) (data not shown).

### A subset of SAM survivors are metabolically distinguishable from community participants.

**Figure 1:**
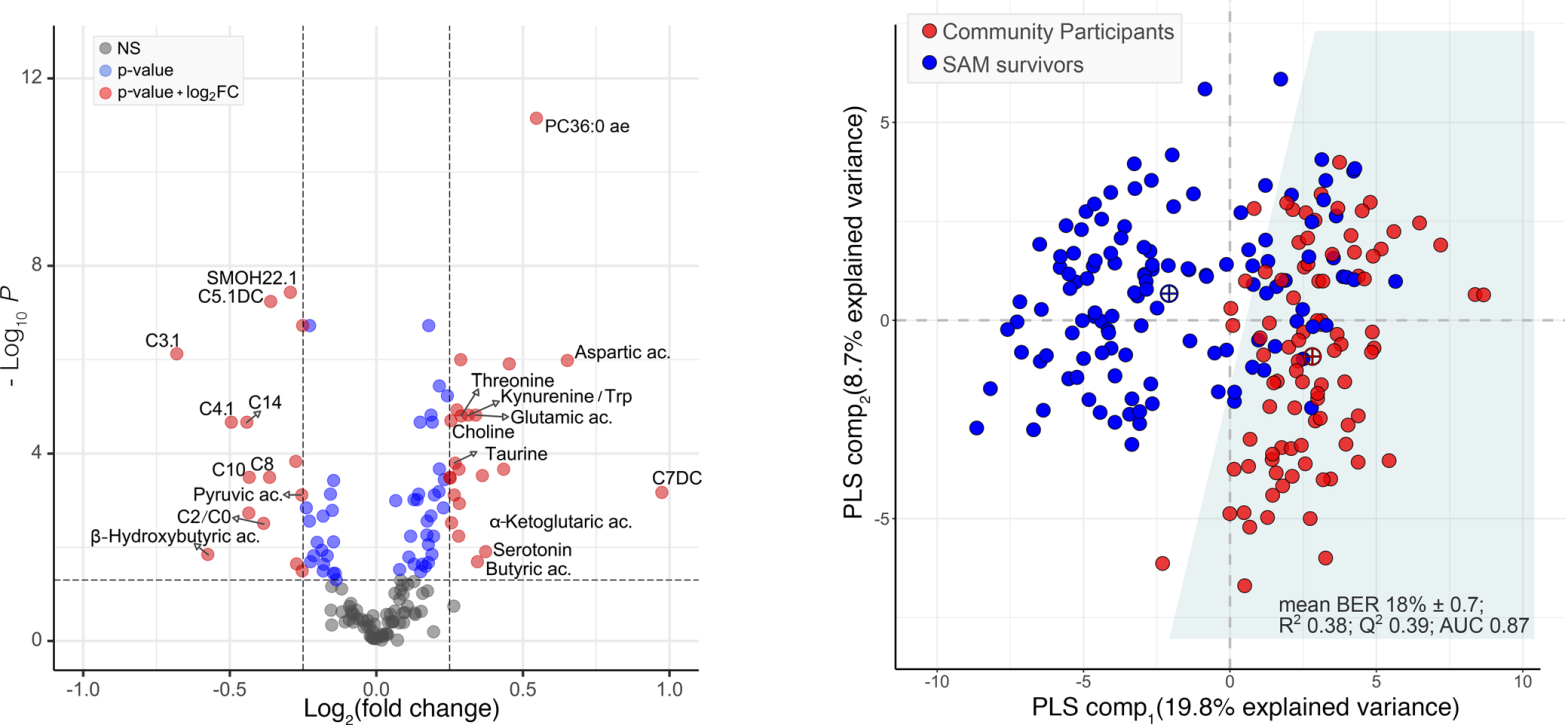
Group classification of adult SAM survivors and age-, sex-, and BMI-matched community participants based on the serum concentrations of metabolite variables. (**A**) Volcano plot displaying the differential metabolite variables measured in serum of either adult SAM survivors (*n* = 122) or community participants (*n* = 90). The horizontal *x* axis plots the log_2_ fold change of metabolite variables between groups, while the vertical *y* axis represents the negative log_10_ FDR-corrected *P* value obtained from linear models testing group differences while adjusting for age, sex, and BMI. Gray dots represent metabolite variables that are nonsignificant (NS); blue dots are those with FDR-corrected *P* < 0.05; red dots are those with both FDR-corrected *P* < 0.05 and a log_2_ fold change between groups > 0.25 or < –0.25. Positive *x* values represent metabolite variables that are higher in adult SAM survivors while negative *x* values represent those that are lower. (**B**) Two-dimensional sparse partial least square determinant analysis (sPLS-DA) score plot showing a partial separation of adult SAM survivors (blue circles) and community participants (red circles) based on the serum concentrations of the top selected metabolite variables. Crossed circles indicate group centroids colored as per legend, and the white versus light blue zones demarcate the decision line for group classification. sPLS-DA was performed on standardized concentrations of metabolites that were Box-Cox transformed and adjusted for age, sex, and BMI. BER, balanced error rate.

**Figure 2:**
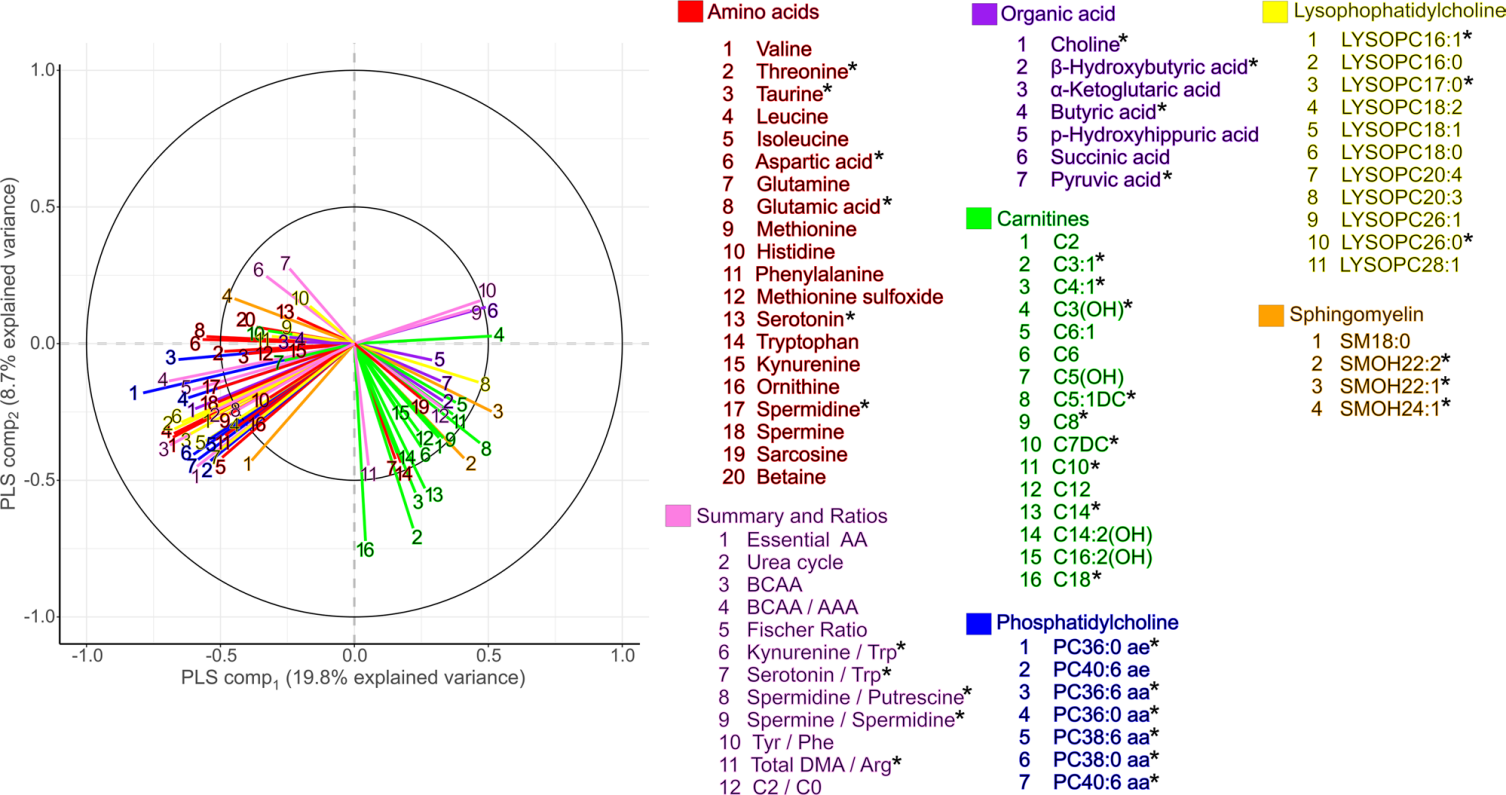
Correlation plot of the 77 retained metabolite variables in serum that best distinguish SAM survivors (*n* = 122) from community participants(*n* = 90) based on sPLS-DA. Asterisks indicate metabolite variables that also have both an FDR-corrected *P* < 0.05 and an absolute log_2_ fold change > 0.25 in univariate models. AA, amino acids; FC, fold change.

Several metabolite variables differed between SAM survivors and community participants, and overall, metabolite profiles could be used to distinguish most SAM cases from those of community participants (Figure 1). While correcting for age, sex, and BMI (and age and sex only), 77 metabolite variables met the feature selection criteria of both being FDR significant in univariate linear models (Figure 1A) and being selected by the sparse PLS-DA models using cross-validation designed to distinguish between SAM survivors and community participants (Figure 1B and Figure 2). Based on permutation testing, group classification by the PLS-DA model was better than random (*P* < 0.001) (Supplemental Figure 1). The mean BER based on centroid distance was 18% ± 0.7%, with an R^2^ = 0.38, and Q^2^ = 0.39 (indicating acceptable consistency between the predicted and original data) (21), while the area under receiver operating characteristic (ROC) curves (AUROCs) for PLS-component_1_ = 0.87. The results were similar when a training/test split of the cohort was used; AUROC was 0.85 (95% CI 0.80–0.91) for the training set with a misclassification error of 14.6%. For the test set, AUROC was 0.83 (95% CI 0.71–0.95) with a misclassification error of 17.4%.

**Figure 3:**
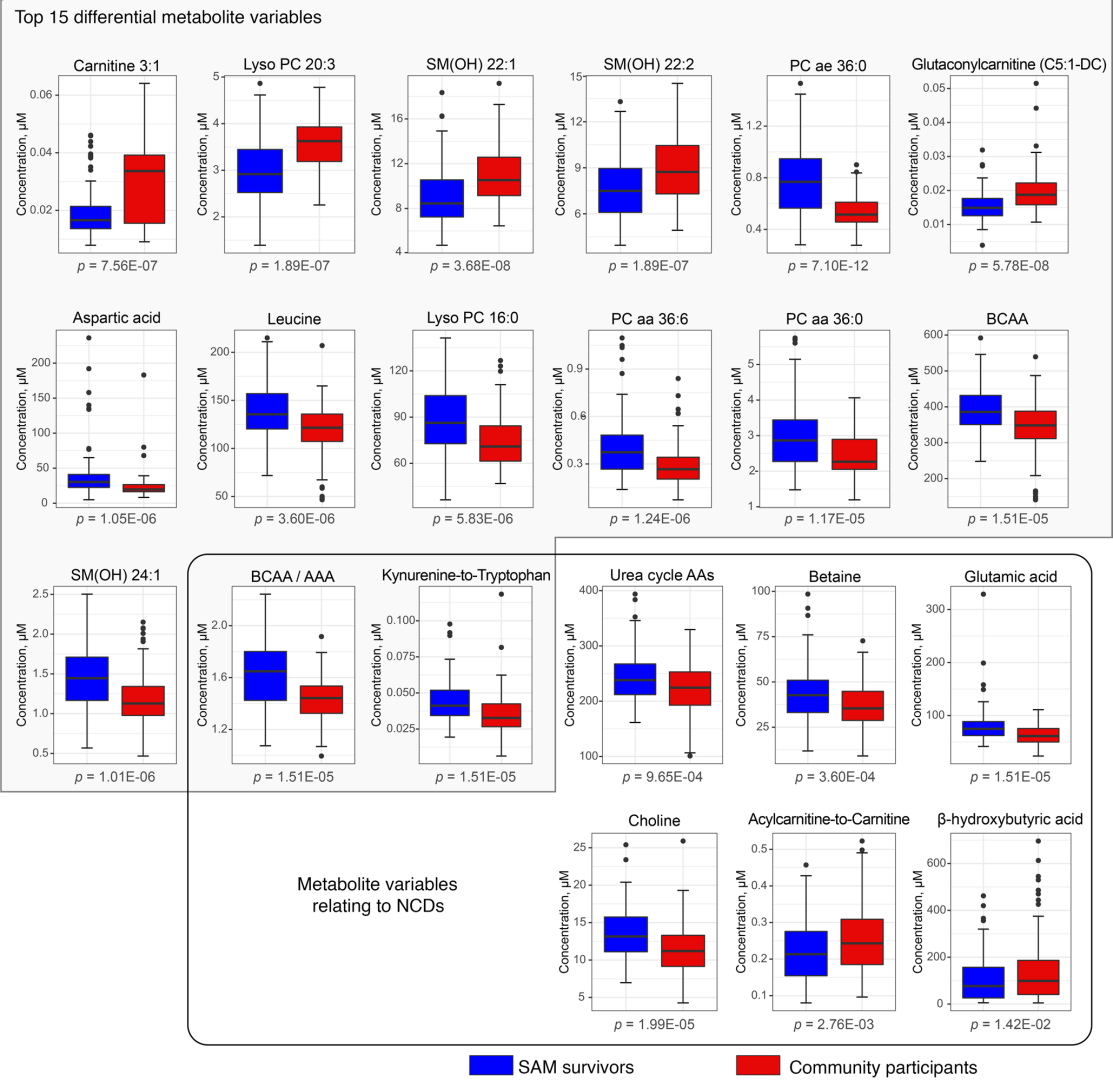
Box plots of differentially expressed metabolite variables in serum from the TMIC PRIME Assay mass spectrometry–based analysis between SAM survivors (*n* = 122) and age-, sex-, and BMI-matched community participants (*n* = 90). Box plots summarize medians (midline) and IQRs; circles represent outlying data points; FDR-corrected *P* values are presented. Gray box includes the top 15 metabolite variables that were most differential between groups; black box highlights differential metabolites previously associated with NCDs. Lyso PC, lysophosphatidylcholines; SM, sphingomyelins, PC aa, phosphatidylcholine di-acyl; PC ae, phosphatidylcholines acyl-alkyl.

Specifically, the 77 differential variables were 16/40 acylcarnitines, 20/22 amino acids, 4/10 sphingomyelins, 11/14 lysophosphatidylcholines, 7/14 phosphatidylcholines, 7/16 organic acids, and 12/20 summary and ratio variables. Results from univariate analyses are detailed in Supplemental Table 2. The top 15 metabolites that best distinguished SAM survivors and community participants based on variable importance in the projection score are listed in Table 2 and presented in Figure 3. As seen in the correlation plot (Figure 2), the mean concentrations of most amino acids, namely leucine, aspartic acid, glutamic acid, valine, and threonine, and related summary values (e.g., total essential amino acids, urea cycle amino acids, BCAAs, BCAA/AAA), were higher in SAM survivors. However, tryptophan was a notable exception as it was lower in adult SAM survivors compared with community participants, and this was linked to SAM survivors having higher KT ratios. Similarly, choline and a subset of phosphatidylcholines, sphingomyelins, and lysophosphatidylcholines — PC ae C36:0, PC aa C36:0, PC aa C36:6, SM(OH) C24.1, lysoPC a 16:0 — were also higher in SAM survivors. In contrast, the mean concentration of most acylcarnitines (including C5:1-DC, C3:1, and C14) and certain sphingomyelins and lysophosphatidylcholines [SM(OH) C22.1, SM(OH) C22.2, and lysoPC a 20:3] were lower in SAM survivors than in community participants. Additionally, the ratio of acylcarnitine to free carnitine (C2/C0), a marker of fatty acid β-oxidation, was lower in SAM survivors, as was β-hydroxybutyric acid. While the overall sparse PLS-DA model suggests that the metabolic profile of many SAM survivors can be distinguished from that of community participants, the observed fold change between groups of individual metabolite variables was very small. Some SAM survivors seemed more readily distinguishable from control participants; however, this potential subgroup was not related to SAM type (i.e., severe wasting vs. edematous malnutrition) (Supplemental Figure 2). Additionally, while roughly 43% of female SAM survivors and 56% of male SAM survivors were below average height for the population (i.e., women < 160.8 cm, men < 171.8 cm), SAM survivors of either sex who were below average height and those who were at or above average height had similar metabolic profiles (data not shown).

### Survivors of SAM have similar metabolic profiles.

**Figure 4:**
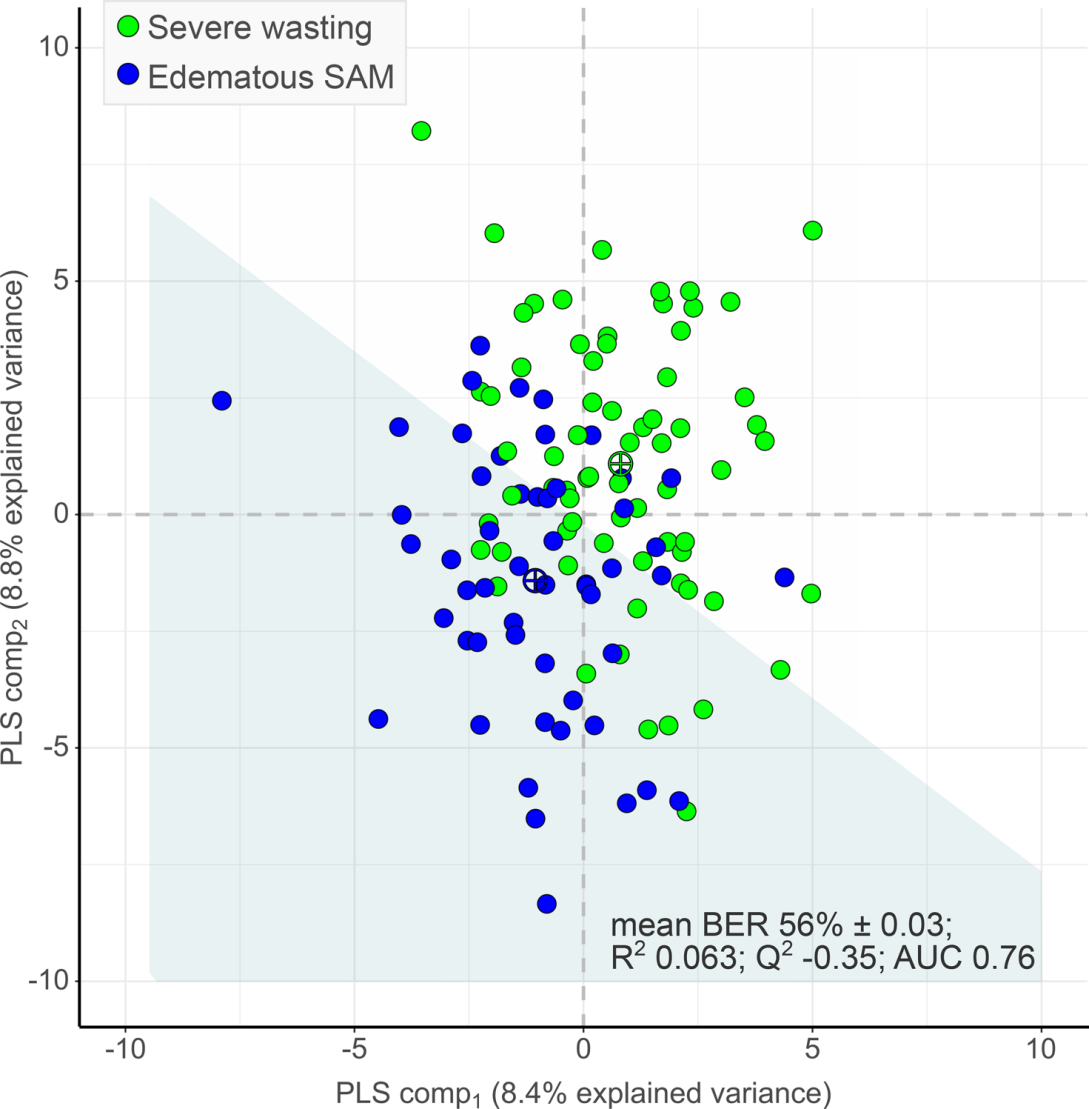
Two-dimensional PLS-DA score plot showing group classification of adult survivors of severe wasting (*n* = 69, green circles) and edematous malnutrition (*n* = 53, blue circles) based on the serum concentrations of measured metabolite variables. Crossed circles indicate group centroids colored as per legend; the white versus light blue zones demarcate the decision line for group classification. PLS-DA was performed on standardized concentrations of metabolites that were Box-Cox transformed and adjusted for age, sex, and BMI.

Our PLS-DA models adjusted for age, sex, and BMI could not distinguish the metabolic profiles of SAM survivors who experienced edematous SAM from those who experienced severe wasting (Figure 4). Permutation testing showed that classification of the 2 SAM phenotypes by the PLS-DA model was not better than random (*P* = 0.71). The mean BER based on centroid distance was 56.3% ± 0.03%, R^2^ = 0.063, Q^2^ = –0.35, and AUC for PLS-component_1_ = 0.76.

Similarly, age-, sex-, and BMI-adjusted univariate linear models did not identify any differential metabolite variable between the 2 SAM phenotypes. Results from generalized linear models additionally adjusted for birth weight or income were also nonsignificant for all metabolites tested (data not shown).

### Specific metabolite variables are associated with cardiometabolic risk factors.

**Figure 5:**
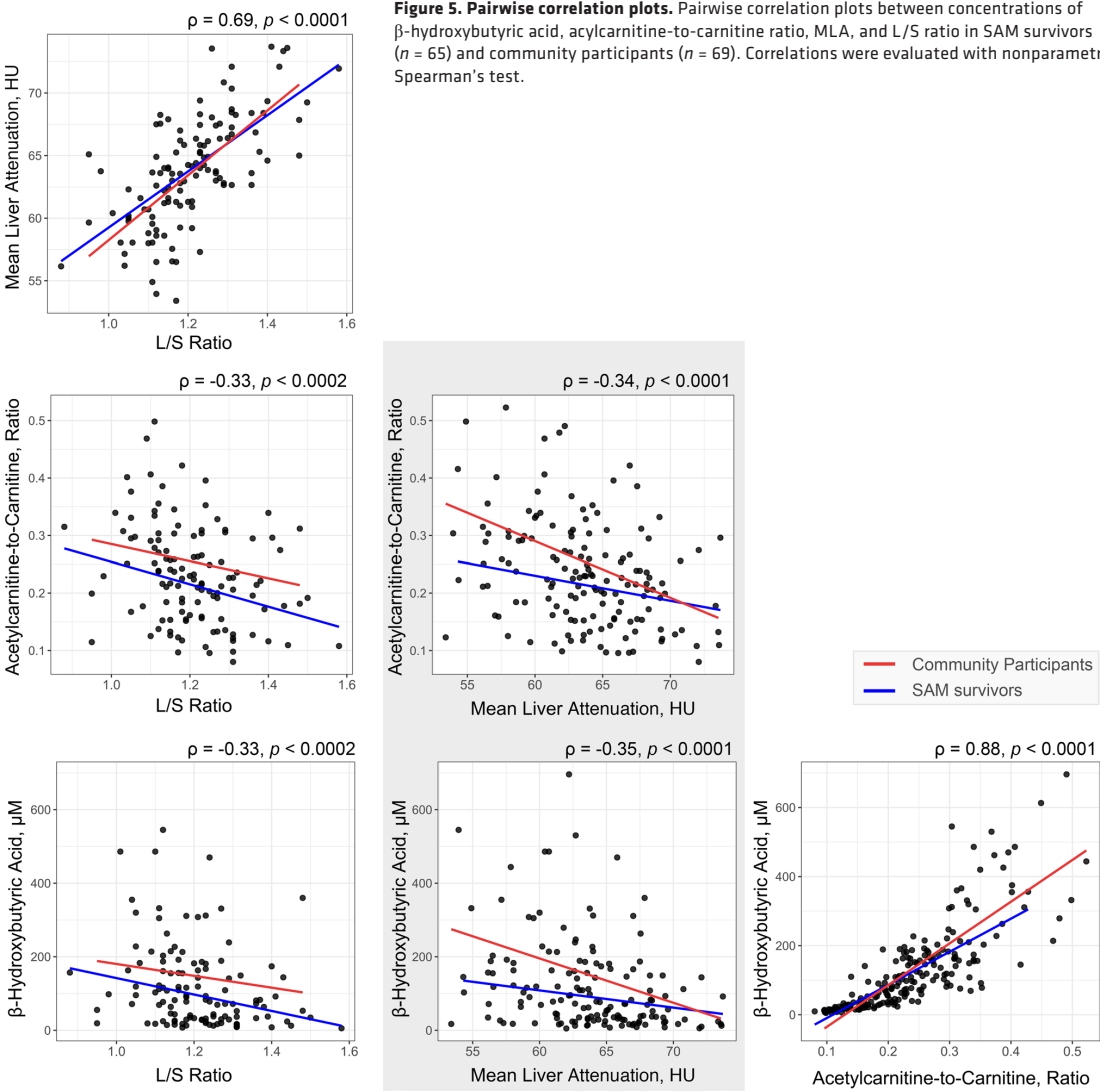
Pairwise correlation plots. Pairwise correlation plots between concentrations of β-hydroxybutyric acid, acylcarnitine-to-carnitine ratio, MLA, and L/S ratio in SAM survivors (*n* = 65) and community participants (*n* = 69). Correlations were evaluated with nonparametric Spearman’s test.

Metabolite variables that differ with SAM exposure and are related to cardiometabolic risk were tested for association with fat mass, mean diastolic pressure, mean systolic pressure, HOMA-IR, WBISI, oDI, and estimates of liver fat while adjusting for age, sex, and BMI. Box plots of these selected metabolite variables (i.e., BCAA/AAA ratio, KT ratio, urea cycle metabolites, choline, betaine, glutamic acid, C2/C0, β-hydroxybutyric acid) are presented in Figure 3. After accounting for multiple testing, we found that both β-hydroxybutyric acid and C2/C0 were associated with measures of liver fat (Figure 5). These variables were highly correlated (*P* = 0.88, *P* < 0.0001) and inversely associated with both MLA (*P* = –0.34, *P* < 0.0001) and L/S ratio (*P* = –0.33, *P* = 0.0002). The interactive effect between SAM exposure and MLA (i.e., difference in slope) was tested but only tended toward significance (interaction term for C2/C0 model, *P* = 0.077; for β-hydroxybutyric acid model, *P* = 0.052, Figure 5). Overall, models explained a relatively modest proportion of variance. The models for β-hydroxybutyric acid versus MLA or L/S ratio both had an adjusted R^2^ of 0.21, but the partial R^2^ for SAM exposure was only 0.060 (in the case of MLA) and 0.049 (in the case of L/S) (Supplemental Table 3). The C2/C0 models were slightly stronger (MLA, adj. R^2^ 0.33; L/S ratio, adj. R^2^ 0.40). However, SAM exposure still explained less than 7% of variance in C2/C0 (partial R^2^ 0.058 and 0.068, respectively) (Supplemental Table 3).

### Participant subclusters are driven by sex, body composition, and metabolites.

**Figure 6:**
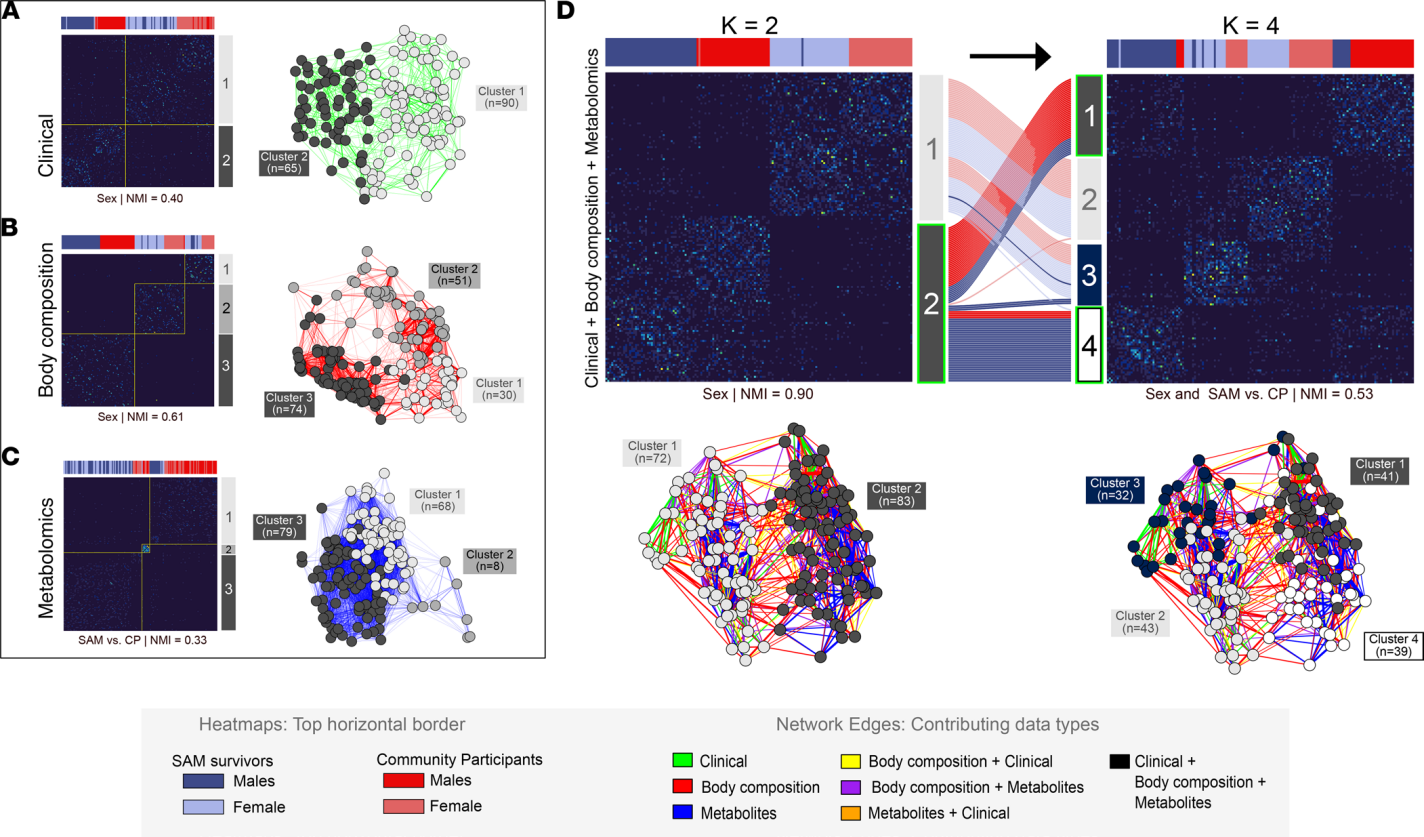
Participant pairwise similarity. Participant pairwise similarity (*n* = 155) presented by heatmaps (left) and networks (right) generated from (**A**) clinical characteristics, (**B**) body composition, (**C**) metabolite variables, and (**D**) all data types integrated by SNF to form 2 or 4 (K = 2 vs. K = 4) clusters of participants based on the spectral clustering of similarity matrices. The alluvial plot illustrates the redistribution of participants classified into either 2 or 4 clusters where each flow line, colored as per legend, represents the redistribution of a participant between groups. Heatmap colors indicate similarity between participants (dark blue, low similarity; progression toward yellow, increasing similarity). Colors in top horizontal border code for participant attributes: SAM survivors (blue) versus community participants (red) and men (dark) versus women (pale). In network plots, nodes (circles) represent participants colored in gray scale by cluster assignment as per vertical border legend on right of heatmaps. Network edges (lines) represent participants’ pairwise similarities: as per legend, colors indicate contributing data type(s), and both edge thickness and length reflect similarity between participant pairs (i.e., long edges connect participants with low similarity, and short edges connect those with high similarity). Normalized Mutual Information (NMI) metrics assess clustering quality by indicating whether cluster assignment reflects known group labels (e.g., sex or SAM exposure); NMI values are between 0 and 1, with 1 indicating perfect cluster alignment with group labels and 0 having no mutual information (i.e., groups are completely split across clusters). CP, community participant; SNF, similarity network fusion.

To further explore participant subclustering in an agnostic manner, we ran a similarity network fusion (SNF) analysis (Figure 6). This method first builds networks of similar participants based on each data set separately and then fuses these single data networks through iterations of spectral clustering. This unsupervised method was used to integrate the 3 data types available (i.e., clinical features, body composition, and metabolite variables) (Supplemental Table 4) and reveal participant subclusters based on the similarity between subjects.

As visualized by the split in dark (male) versus pale (female) colors in the top horizontal border of heatmaps, the clusters obtained from clinical features (Figure 6A) and body composition (Figure 6B) mostly aligned with sex (NMI, 0.40 and 0.61, respectively). Body composition clusters were mainly associated with measures of adiposity (truncal fat mass, android fat mass, and total fat mass; *P* < 0.001) and lean mass (total lean mass and lean mass in trunk, leg, and arm compartments; *P* < 0.001). However, while men mostly grouped together in body composition cluster 3 (BMI 21.9 ± 2.5; fat mass 6.3 kg ± 4.2; lean mass 56 kg ± 6.0), women were further split into 2 subclusters that differed mainly by measures of adiposity, where cluster 1 (*n* = 30) was composed of lean individuals (BMI 19.5 ± 2.2; fat mass 10.6 kg ± 4.8; lean mass 34.5 kg ± 4.8) while cluster 2 (*n* = 51) mostly grouped women that tended toward overweight and obesity (BMI 28.4 ± 5.1; fat mass 30.4 kg ± 10.7; lean mass 41.0 kg ± 6.4, *P* < 0.001). Similarity clustering based on metabolite variables generally split SAM survivors versus community participants (blue vs. red, NMI, 0.33); the small subcluster of 8 participants identified (Figure 6C) is possibly related to deviations from the study protocol such as incomplete fasting. The integration of all data types (Figure 6D) revealed that the 2 most prominent participant clusters (K = 2) were strongly related to sex (NMI 0.90, *K = 2 cluster 1*: *n* = 72, female 99% [light gray nodes]; *K = 2 cluster 2*: *n* = 83, 99% males [dark gray nodes]). The long separating edges (red and yellow) between these clusters were mostly related to body composition. When participants were grouped into 4 clusters (K = 4), the NMI was 0.53 for the groups cross-split by both sex and SAM exposure. However, as seen in the alluvial plot, which traces how individuals flow between clusters split, the women-dominated groups contained a mix of survivors and community participants, with *K = 4 cluster 2* (*n* = 43) containing 49% female cases and 51% community participants (light gray nodes) and *K = 4 cluster 3* (*n* = 32) containing 53% female cases and 34% community participants (blue nodes). However, the men-dominated group (*K = 2 cluster 2*) split along SAM exposure as *K = 4 cluster 4* (*n* = 39) contained 87% male survivors (white nodes) while *K = 4 cluster 1* (*n* = 41) contained 78% male community participants (dark gray nodes). Also, the similarity grouping subclusters tended to be driven by metabolic features, as illustrated by for example the tight net of short blue edges between nodes of *K = 4 cluster 1* and *K = 4 cluster 4*. Thus, subclusters associated with having experienced SAM in childhood were more evident in males.

## Discussion

This study is one of the first to investigate the metabolic profiles of adults who were hospitalized with severe malnutrition in early childhood using targeted metabolomic analyses. As we hypothesized, the metabolic profiles of adult SAM survivors differed from community participants, and several of the distinguishing metabolite variables had recognized associations with cardiometabolic risk factors.

We report that Jamaican adult SAM survivors (age 28.4 ± 8.8 years, >20 years after hospital discharge) showed differences from community participants of overall similar age, BMI, and body composition in 77 metabolite variables measured in fasting serum. The profile differences were related to increases in most amino acids but not tryptophan; increases in choline and certain phosphatidylcholines, sphingomyelins, and lysophosphatidylcholines; and decreases in many acylcarnitines in SAM survivors compared with community participants. It is to be noted, however, that the observed fold change of individual metabolite variables was very small in the fasted state. These differences might be amplified with age or after a metabolic challenge. Additionally, some SAM survivors were more readily distinguishable from community participants than others and might thus be more vulnerable to cardiometabolic risk.

In contrast, we have previously shown that younger Malawian SAM survivors (aged 9.6 ± 1.6 years, 7 years after hospital discharge) did not show differences in their metabolic profiles compared with community and sibling participants (14). Thus, the metabolic signatures linked to NCDs that we describe may start to manifest as SAM survivors age. Our cohort is itself relatively young in terms of developing NCDs, yet differences that could set adult SAM survivors on a potentially unfavorable health trajectory were already detected. Differences between the 2 settings could also be linked to factors other than age, such as specific environmental exposures (pollution, water quality, obesity, and adult dietary patterns) or differences in diagnostic criteria and treatment strategies. Additionally, in our previous study, more than 15% of the children were HIV-positive and more than 31% had unknown HIV status, whereas HIV-positive individuals were excluded from this current study.

Our secondary hypothesis was that survivors of severe wasting would have a distinct metabolic profile from survivors of edematous malnutrition, especially given that severe wasting is associated with lower birth weight (22). However, while survivors of severe wasting had lower BMI, waist circumference, lean mass, fat mass, and android fat than survivors of edematous malnutrition, metabolic differences were not found between survivors of these 2 phenotypes. It is notable that although these young adult SAM survivors were not overweight generally (mean BMI < 25 kg/m^2^), survivors of severe wasting had lower lean muscle mass. Thus, the observed changes in body composition between SAM phenotypes might not be sufficient, at this stage, to differentially influence their metabolic profiles in a way that can be detected with a static measure of fasting serum. However, survivors of severe wasting could still be at greater long-term risk, especially considering the link between reduced lean muscle mass and the development of NCDs later in life (23, 24). Additionally, we acknowledge that the effects of aging and/or a metabolic challenge in this group will be important to evaluate in future studies because many of these young and mostly lean participants might still either be suffering from low nutrition quality (at worst) or not be exposed to a sufficiently obesogenic diet.

### The association between SAM exposure and adult body size and composition is sex specific.

We report an interaction between sex, SAM exposure, and adult anthropometry and body composition. Male SAM survivors showed a “small” phenotype, being of shorter stature, weighing less, and having less lean mass, while having a greater AG fat ratio than males from the community. This finding might have intrauterine origins as boys grow faster than girls from an early stage of gestation, and this makes them more vulnerable if their nutrition is compromised (25). Greater AG fat ratio and reduced lean mass in male SAM survivors might have important clinical and metabolic implications, as these factors are associated with an increased risk for metabolic syndrome in healthy adults (26). Additionally, they might also be at risk for later sarcopenic obesity. In keeping with the idea of greater long-term risk in male SAM survivors, Mexican men who experienced malnutrition in their first year of life were shown to be more glucose intolerant and hyperinsulinemic compared with controls, using OGTT (17). While female SAM survivors did not differ in body composition from females from the community, clustering analysis revealed that women may show more diverse body type subgroups, which could mask the effects of SAM exposure. Also, this divergent sex effect could be due to BMI representing slightly different body composition in men (muscle mass per unit height) versus women (fat mass per unit height). These differences, particularly in height, lean mass, and body fat distribution, could impose an additional cardiometabolic risk particularly in male SAM survivors, especially if they become obese in later life.

### Metabolic profiles in relation to risk of T2D.

We questioned specifically whether metabolic perturbations linked to having experienced SAM in early childhood could persist and/or be associated with the cardiometabolic risk profiles of adult survivors.

As previously demonstrated (19), some members of this cohort of adult SAM survivors had similar insulin sensitivity and β cell function to community participants. However, this subset of SAM survivors had higher concentrations of BCAAs and AAAs, 5 of which (isoleucine, leucine, valine, tyrosine, and phenylalanine) have reported associations with diabetes risk in normoglycemic individuals (27). Additionally, adult SAM survivors had lower tryptophan and an associated higher KT ratio, which has been identified as a predictor of incident T2D and coronary events, with the dysregulation of the KT metabolic pathway described as one of the mechanisms of insulin resistance (28). Further, SAM survivors had higher median concentrations of urea cycle amino acids (arginine, citrulline, ornithine, aspartic acid, and urea), which have been associated with T2D (29, 30). The higher glutamic acid seen in SAM survivors has also been associated with both increased 2-hour plasma glucose and higher tertiles of HOMA-IR (31).

Furthermore, 2 phosphatidylcholine subclasses (diacyl and acyl-alkyl phosphatidylcholines) were higher in SAM survivors than community participants, with PC ae C36:0 showing the greatest difference. These structural lipids (i.e., constituents of cell membranes) are also involved in cell signaling and metabolic control (32), and together with other choline-containing phospholipids, such as lysophosphatidylcholines and sphingomyelins, have been linked to increased risk of T2D (33). Some studies report lower acyl-alkyl-phosphatidylcholines in subjects with insulin resistance (34), but these results were not replicated in our cohort.

Taken together, these metabolic findings could suggest greater risk of glucose dysmetabolism and eventual T2D in SAM survivors, albeit in the current absence of overtly impaired insulin sensitivity or clinical disease that may develop with obesity and age based on oral glucose tolerance test (OGTT) (35).

### Metabolic profiles in relation to risk of fatty liver disease.

The hydrolysis of lipid stores and the oxidation of fatty acids are key acute adaptive responses to SAM, evidenced by high circulating levels of free fatty acids (FFAs), ketones, and even-numbered acylcarnitines (36). Ultimately, in both SAM phenotypes, hepatic mitochondrial function is reduced and associated with hepatic steatosis (37, 38). Although hepatic fat does accumulate during a SAM episode, it resolves completely (albeit slowly) with recovery (39). However, it is unclear whether any lingering metabolic perturbations could affect later hepatic fat metabolism.

Nonalcoholic fatty liver disease (NAFLD) results from either excess FFA delivery from diet or peripheral stores (secondary to peripheral insulin resistance) or decreased intrahepatic FFA oxidation and increased de novo lipogenesis (40). While SAM survivors had less liver fat than community participants, the difference was small, and both groups failed to meet the criteria for moderate-to-severe fatty liver (L/S < 1). However, compared with community participants, SAM survivors had lower β-hydroxybutyric acid concentrations and lower C2/C0 (related to lower acylcarnitine, C2, concentrations). C2 and β-hydroxybutyric acid were highly correlated in our data and are both known to regulate β-oxidation of FFA. β-hydroxybutyric acid is a marker of hepatic ketogenesis after FFA oxidation, and its production rate is the major determinant of its concentration in serum (41, 42). Carnitine is essential for the uptake of fatty acids into mitochondria prior to β-oxidation and is known to be reduced in persons with NAFLD (43). Interestingly, the associations between liver fat and both β-hydroxybutyric acid and C2/C0 are weaker in SAM survivors compared with community participants, suggesting that the diagnosis of SAM might somehow diminish the association between these metabolites and liver fat.

These metabolic findings suggest that while early life SAM may limit β-oxidation, at this stage, the impact on the development of NAFLD is inconclusive. This is consistent with a recent study in rodents where protein restriction after weaning and subsequent feeding of a high-fat and -carbohydrate diet did not induce hepatic steatosis (44). However, decreased fatty acid oxidation in SAM survivors could represent a harbinger for later hepatic steatosis in these currently still young, lean SAM survivors. Additionally, the timing and severity of the early nutritional insult (prenatal vs. postnatal) might variably influence the development of NAFLD, and, because intrauterine growth restriction has been associated with subsequent NAFLD (45), the combined insults may be additive. Changes in metabolites other than those related to β-oxidation could also potentially affect the risk of developing NAFLD. For example, the higher KT ratio seen in SAM survivors might be evidence of defective indoleamine 2,3-dioxygenase activity, and this has been associated with liver inflammation and fibrosis (46).

### Metabolic evidence of risk of hypertension.

SAM survivors and community participants had similar systolic and diastolic blood pressure. Although 20.8% of our participants had elevated blood pressure readings (21.3% with measured diastolic blood pressure ≥ 80 mmHg, 3.9% with measured systolic blood pressure ≥ 140 mmHg), we did not demonstrate a significant association between any targeted metabolite and elevated systolic blood pressure, elevated diastolic blood pressure, or a combination of the two. However, choline and choline-containing molecules have a reported association with the development of hypertension (47), and both choline and phosphatidylcholine concentrations were higher in SAM survivors compared with community participants. Several other metabolites with reported associations with elevated blood pressure ([alpha]-1 acid glycoproteins, ref. 47; and serum free fatty acids, i.e., heptanoic, oleic, nonanoic, eicosanoic, and hexanoic acids, ref. 48) were not targeted for analysis in this study; thus, our findings may be inconclusive.

### Limitations and strengths.

This study has limitations. First, although representative of the full cohort based on age, sex, and BMI, the number of participants analyzed here included less than half of those enrolled in the main cohort; thus, certain subanalyses might be underpowered. Additionally, CT scans are less reliable at evaluating mild liver fat accumulation. Also, metabolites measured at fasting must be interpreted with some caution because their concentrations do not reflect pathway flux (increased production versus decreased utilization). Missing data in certain variables (e.g., birth weight for community participants, specific dietary information, income, and L/S ratio) made it difficult to account for additional suspected confounders and/or include all participants for certain analyses, such as SNF. Despite these limitations, the study was strengthened by several factors: (a) the cohort is very well characterized with rich longitudinal clinical data, (b) we ensured high analytical sensitivity by using targeted mass spectrometry and measuring serum as opposed to plasma, and (c) data were analyzed using conventional statistical approaches with both supervised and unsupervised machine learning methods for feature selection, controlling FDRs, mitigating overfitting, and describing general patterns. Importantly, we demonstrated for the first time to our knowledge that early life SAM could lead to metabolic derangements more than 20 years later, and these may be tracked over time to identify patients at particular risk of developing NCDs.

### Conclusions.

This study is the first to our knowledge to investigate the metabolic profiles of adults who were hospitalized for severe acute malnutrition in early childhood. Our data provide evidence that metabolic profiling can distinguish adult survivors of SAM from unexposed participants living in the same communities and of similar age, sex, and BMI. Some metabolite variables that are greater in adult SAM survivors are associated with the risk of T2D and may be signatures of reduced hepatic fatty acid oxidation and possibly NAFLD. These findings should be validated further using stable isotopes that can capture pathway flux, by exposing SAM survivors to specific metabolic challenges and by repeating studies in older cohorts. Our findings support the hypothesis that persons exposed to SAM in early life have long-term metabolic consequences that could affect risk for NCDs, and thus, they may benefit from targeted clinical case management.

## Methods

### Study design/subjects.

**Figure 7:**
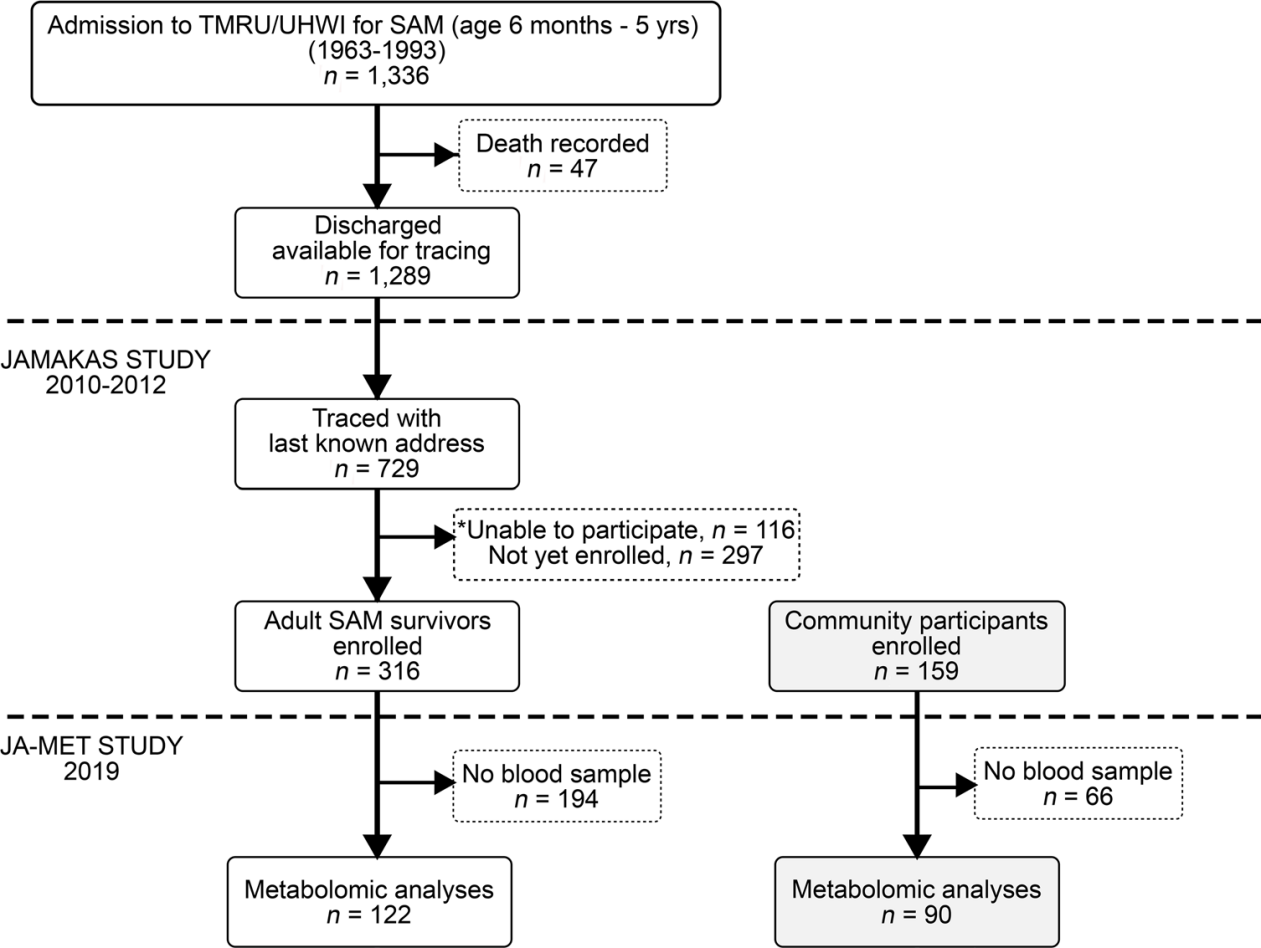
Flow chart detailing recruitment of adult survivors of SAM (*n* = 122) and community participants (*n* = 90). “Unable to participate” includes adult survivors of SAM who were unavailable because of migration (*n* = 53), illness (*n* = 19), refusal (*n* = 14), or pregnancy (*n* = 30). TMRU, Tropical Metabolism Research Unit; UHWI, University Hospital of the West Indies; JAMAKAS, Jamaica Marasmus and Kwashiorkor Adult Survivors; JA-MET, Jamaica Metabolomics.

This is a secondary analysis of the JAMAKAS Study, a cohort study that selected participants based on exposure to SAM in early childhood (detailed flow chart presented in Figure 7). SAM diagnosis was based on the Wellcome criteria (49), the most widely used classification for malnutrition up to 1999. Children presenting with a weight-for-age of 60%–80% and edema were diagnosed with kwashiorkor, here referred to as edematous malnutrition, while those with a weight-for-age less than 60% without edema had marasmus, here referred to as severe wasting (49).

### Selection of adult SAM survivors for metabolomics analysis.

The cohort was assembled by reviewing the records of all 1336 patients admitted to the Tropical Metabolism Research Unit of the University Hospital of the West Indies between the years 1963 and 1993 with a diagnosis of SAM at age 6 months to 5 years (22). With an inpatient mortality rate of 3.5%, a total of 1289 surviving adults from the cohort were theoretically available for tracing. Using the last recorded address and name of parents, community health aides and nurses were able to identify a current address for 729 adult SAM survivors; the remaining 560 members of the cohort were not traced. We excluded all persons with an acute illness, using glucocorticoids, with a known history of a hemoglobinopathy, or who were pregnant or lactating or unable to give written informed consent. Of the 729 persons traced, a further 116 were unavailable to the study because of refusal (*n* = 14), illness (*n* = 19), migration (*n* = 53), and pregnancy (*n* = 30), leaving 613 persons available for recruitment. Of these, 316 adult SAM survivors enrolled in the JAMAKAS Study, and a subset of 122 SAM survivors submitted appropriate fasting blood samples for metabolomic analysis in the JA-MET study (Figure 7).

### Selection of community participants for metabolomics analysis.

Community participants were purposefully selected to be from the same socioeconomic group as SAM survivors. Community health aides recruited 159 participants from within the same communities where each adult SAM survivor lived as follows: starting on the street where the SAM survivor lived, visits were conducted house to house alternately on either side of the road. If unsuccessful, adjacent streets were similarly visited. Height and weight were measured in the field using a stadiometer and a digital scale that was calibrated daily. Community participants were matched based on age ± 5 years, sex, and BMI ± 2 kg/m^2^ that fell within the same BMI class as the SAM survivor being matched (i.e., underweight, less than 18.5 kg/m^2^; normal, 18.5 to 24.9 kg/m^2^; overweight, 25 to 29.9 kg/m^2^; and obese, greater than 30 kg/m^2^). Like SAM survivors, community participants were asked about their general health status using a standardized questionnaire (50). Additional exclusion criteria for community participants were a reported history of SAM and being related to a SAM participant within the study. A total of 90 community participants submitted fasting blood samples for metabolomic analysis in the JA-MET study.

### Measurements.

All assessments and measurements were done contemporaneously in both SAM survivors and community participants as follows.

### Anthropometry.

Body weight was measured to the nearest 0.1 kg using a portable scale (Seca 770). Using a minimum of 2 readings, height was measured to the nearest 0.1 cm using a stadiometer (Invicta) with the participant’s head held in the Frankfurt plane. Waist circumference was measured to the nearest 0.1 cm using a standardized protocol, i.e., at the midpoint between the iliac crest and the lowest rib with the participant standing erect (51).

### Body composition.

Dual x-ray absorptiometry using a Lunar Prodigy machine (GE Healthcare) was used to perform a whole-body scan on each participant. Regions of interest (ROIs) were demarcated to obtain percentage body fat, fat mass, and lean tissue for total body and regional body compartments.

### Liver fat.

Liver fat was estimated with established criteria validated by other groups using liver biopsy as the gold standard (52–54). Specifically, using a Phillips Brilliance 64-slice scanner, a single cross-sectional 5-mm-width CT scan (of 120 kVp, 100 mA) at the intervertebral disc space between T12 and L1 was obtained to image both the liver and the spleen. Data obtained from CT scans were analyzed using E-Film software. Three ROIs were placed on the liver (posterior right lobe, anterior right lobe and left lobe), and 1 ROI was placed on the spleen. Each ROI measured a minimum of 1 cm^2^ and excluded all major blood vessels. ROI attenuation was documented in HU. MLA was calculated using ROIs in the liver, and the L/S ratio was calculated using ROIs in the liver and the spleen. MLA ≤ 40 HU and L/S ratio ≤ 1 both indicate moderate-to-severe fatty liver.

### Intra-abdominal fat.

A second abdominal CT scan was taken between the L4 and L5 intervertebral disc space perpendicular to the scan table, which represented the abdominal area midway L3 and L4. Images taken from the CT scanner were analyzed with the Tissue Composition Module Beta 1.0 software package (Mindways). On each CT image, total adipose area (TAA) and visceral adipose tissue (VAT) were measured by QCT Pro (55). Subcutaneous adipose tissue (SAT) was calculated as follows: TAA – VAT = SAT.

### Oral glucose tolerance test.

After a 10- to 12-hour overnight fast, participants underwent a 75 g OGTT. At 0, 30, 60, 90, and 120 minutes, 5 mL of blood was taken through an antecubital cannula into chilled fluoridated and heparinized tubes for plasma glucose and insulin measurements. The following indices were calculated:

HOMA-IR = (I_0_ × G_0_)/22.5, where G_0_ and I_0_ reflect basal (fasting) glucose and insulin in SI units (56).

WBISI = 10,000/(G_0_ × I_0_ × G_m_ × I_m_)^0.5^, where G_0_ and I_0_ reflect basal glucose and insulin and G_m_ and I_m_ the mean concentrations of glucose and insulin during OGTT.

Insulin secretion was estimated using IGI = (I_30_ – I_0_)/(G_30_ – G_0_), where I_30_ and I_0_ are insulin concentrations at 30 and 0 minutes and G_30_ and G_0_ are glucose concentrations at 30 and 0 minutes.

oDI, pancreatic β cell function adjusted for insulin sensitivity = IGI × WBISI.

### Targeted metabolomic profiling.

Participants were asked to avoid strenuous exercise and alcohol/caffeine intake the day before testing and were fasted overnight for 10–12 hours. Blood samples were collected between 8:30 and 9:30 am and centrifuged (Allegra 6R centrifuge) for 10 minutes at a relative centrifugal force of 1711*g* at 4°C within 30 minutes of collection. Serum specimens were immediately stored at –80°C for later metabolomic analysis. Metabolomic analyses were conducted at TMIC at the University of Alberta, Edmonton, Canada. Samples were screened for all 143 metabolites included in the TMIC PRIME Assay. This targeted quantitative metabolomics approach was applied using both DIMS and reverse-phase LC-MS/MS performed on an API4000 Qtrap tandem mass spectrometer (Applied Biosystems/MDS Analytical Technologies). This assay was used for the targeted identification and quantification of several endogenous metabolites, including biogenic amines (*P* = 20); amino acids (*P* = 21); glycerophospholipids (*P* = 34) including lysophosphatidylcholines (*P* = 15), sphingomyelins (*P* = 10), and phosphatidylcholines (*P* = 9); acylcarnitines (*P* = 40); organic acids (*P* = 17); monosaccharides (*P* = 1); histidines (*P* = 2); and others (*P* = 4). The method combined the derivatization and extraction of analytes and the selective mass spectrometric detection using multiple reaction monitoring pairs. Isotope-labeled internal standards and other internal standards were used for metabolite quantification. Data analysis was done using Analyst 1.6.2.

Each identified metabolite was included in the data analysis if it passed the following quality control cutoffs: (a) a mean coefficient of variability < 25% across experimental batches, (b) >90% detected values, and (c) ≥LOD in at least 50% of either study group (i.e., adult SAM survivors or community participants). If a specific metabolite measure was below the detection range, the value was replaced by half the LOD of that metabolite.

### Statistics.

The sample size was convenience based, restricted by the number of stored serum samples from the cohort. The primary analysis aimed to compare the metabolomic profiles of adult SAM survivors and community participants. Secondary analyses were conducted to (a) compare the metabolite profiles of adult survivors who had experienced edematous malnutrition versus severe wasting and (b) relate the metabolic profiles to markers of T2D, hypertension, and fatty liver disease.

PCA was used on standardized values to detect sample outliers and examine inherent clustering and correlations. To assess differences in metabolite profiles, we conducted both univariate and PLS multivariate analyses. Generalized linear regression models were performed on Box-Cox–transformed variables while adjusting for age, sex, and BMI. Additional models were also run with further adjustment for income. Model fit was assessed through inspection of residuals, and *P* values were corrected for multiple testing using Benjamini-Hochberg FDR. An FDR-adjusted *P* < 0.05 was considered significant. The mixOmics package was used to perform sparse PLS-DA or sparse PLS regression to reveal the multivariate relationships between metabolites and their association to either participant groups or clinical features (57). These methods implement feature selection through ℓ^1^ regularization (LASSO, ref. 58) where top discriminative or associated features can be ranked. For this analysis, metabolites were Box-Cox transformed and adjusted for age, sex, and BMI using linear regression and using model residuals for downstream analyses. Model performance was assessed with quality assessment statistic (Q^2^) ≥ 0.4, BER < 30%, and AUROC > 0.7. SNF analyses were conducted as implemented by the SNFtool package in R (59). Participant similarity networks were constructed for each of the data types; then these networks were combined into a single network that highlights the common network signals (59).

All analyses were done in the full cohort while adjusting for age, sex, and BMI and additionally for income and stratified by sex. Data were analyzed using R Statistical Software (version 3.5.1).

### Study approval.

This study was conducted according to the guidelines laid down in the Declaration of Helsinki. All procedures involving human subjects were approved by The University of the West Indies Ethics Committee (reference: ECP 34, 17/18) and the Hospital for Sick Children Research Ethics Board (REB: 1000059930). Written informed consent was obtained from all participants.

## Author contributions

DST, MSB, TEF, GBG, and RHJB designed the research; DST conducted data collection and coordinated serum sample transfer. CB, DST, PM, GBG, and RHJB analyzed the data, DST wrote the first draft of the manuscript, RHJB and CB provided main edits of content, and RHJB had responsibility for final content. All authors read and approved the final manuscript.

## Supplementary Material

Supplemental data

ICMJE disclosure forms

## Figures and Tables

**Table 2:**
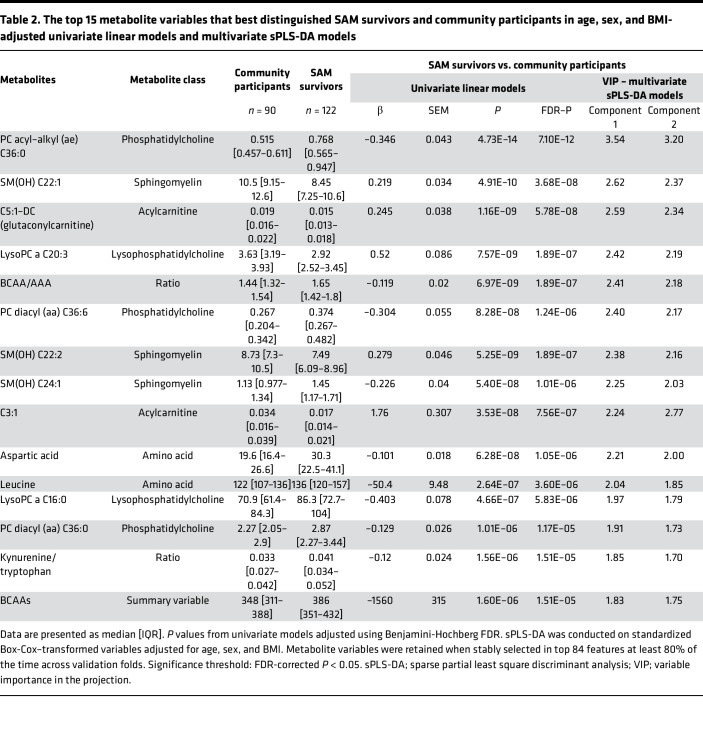
The top 15 metabolite variables that best distinguished SAM survivors and community participants in age, sex, and BMI-adjusted univariate linear models and multivariate sPLS-DA models

**Table 1:**
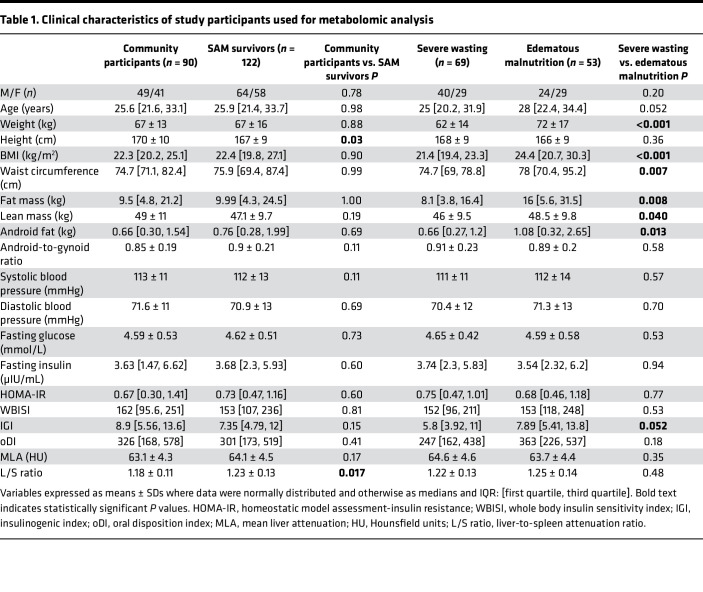
Clinical characteristics of study participants used for metabolomic analysis

## Version 1 11/17/2020

In-Press Preview

## Version 2 12/17/2020

Electronic publication

## Version 3 03/08/2024

The title was updated
