# Supplementary material for: Childhood severe acute malnutrition is associated with metabolomic changes in adulthood
Source: JCI Insight. 2020 Nov 17;5(24):e141316. doi: 10.1172/jci.insight.141316 (PMC7819749; doi:10.1172/jci.insight.141316)
Supplement: Supplemental data [file jciinsight-5-141316-s010.pdf]

Supplementary Figure 1

Discrimination between classes of PLS-DA models based on permutation class-assigment compared to true classification

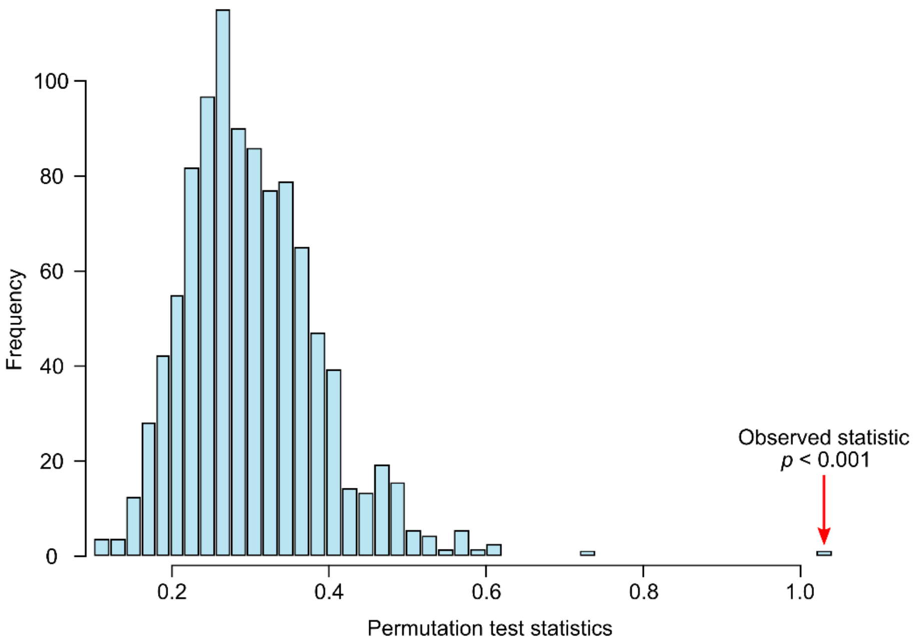

Supplementary Figure 1: Permutation test; Select test statistic: Separation distance (B/W), set permutation numbers:100, $p < 0.001$

Supplementary Figure 2

Two dimensional sparse PLS-DA score plot showing partial separation of adult SAM survivors not to be related to SAM type

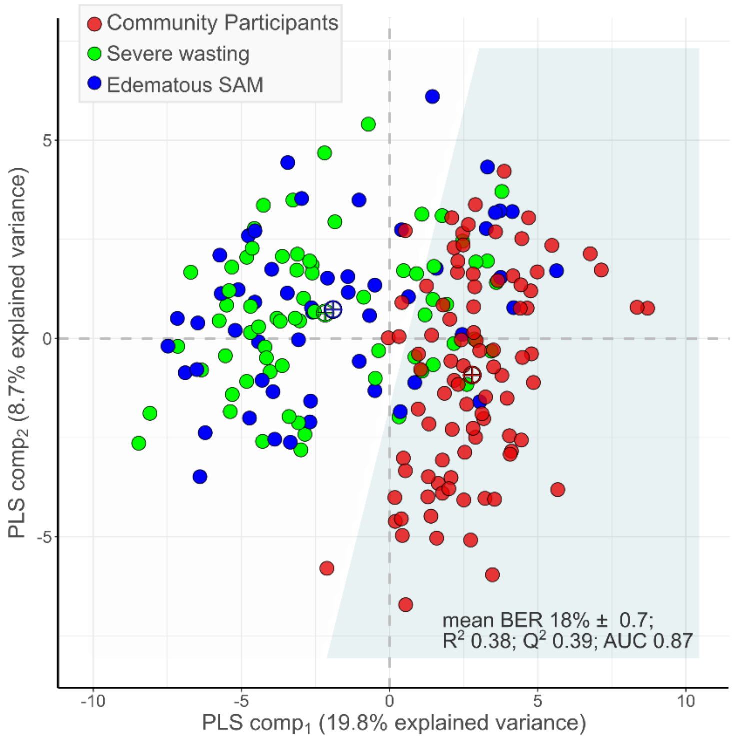

Supplementary Figure 2: Two dimensional sparse PLS-DA score plot showing that partial separation of adult SAM survivors (n = 122, blue and green circles) from community participants (n = 90, red circles) is not related to SAM type (i.e., severe wasting (n = 69, green circles) vs. edematous malnutrition (n = 53, blue circles) based on the serum concentrations of the top selected metabolite variables. Crossed circles indicate group centroids as per figure legend and the white vs. light grey zones demarcate the decision line for group classification between SAM and community participants. Sparse PLS-DA was performed on standardized concentrations of metabolites that were Box-Cox-transformed and adjusted for age, sex, and BMI. AUC, area under the receiver operating characteristic curve; BER, balanced error rate; PLS-DA, partial least-squares discriminative analysis; SAM, severe acute malnutrition.

Supplementary Table 1: Clinical characteristics of 113 male and 99 female study participants used for metabolomic analysis

	Men			Women		
	**SAM Survivors (n = 64)**	**Community Participants (n = 49)**	***p*-value**	**SAM Survivors (n = 58)**	**Community Participants (n = 41)**	***p*-value**
**SBP (mmHg)**	111.9 ± 12.7	113.3 ± 8.8	0.49	111.8 ± 13.0	111.6 ± 14.0	0.99
**DBP (mmHg)**	69.76 ±11.6	72.10 ± 10.1	0.26	72.3 ± 13.6	71.0 ± 12.3	0.63
**Height (cm)**	170.6 ± 7.8	176.0 ± 7.1	<0.001	163.2 ± 7.4	161.7 ± 5.6	0.63
**Weight (kg)**	63.6 ± 12.5	68.1 ± 9.4	0.03	70.0 ± 19.3	65.2 ± 15.9	0.21
**BMI (kg/m²)**	21.8 ± 3.5	22.0 ± 2.5	0.67	26.5 ± 7.2	24.9 ± 5.6	0.22
**Waist (cm)**	74.6 ± 7.8	75.5 ± 6.9	0.53	84.6 ± 17.6	81.3 ± 14.6	0.31
**Fat Mass (kg)**	6.9 ± 6.4	7.3 ± 5.5	0.72	26.3 ± 14.9	23.4 ± 12.3	0.3
**Lean Mass (kg)**	53.5 ± 7.8	57.5 ± 6.6	0.003	39.9 ± 5.8	38.5 ± 4.6	0.2
**Android:Gynoid Fat**	0.92 ± 0.2	0.85 ± 0.2	0.047	0.88 ± 0.2	0.86 ± 0.2	0.73
**MLA (HU)**	64.6 ± 4.1	63.6 ± 5.0	0.31	63.6 ± 4.9	62.7 ± 3.4	0.37
**LS Ratio**	1.23 ± 0.1	1.17 ± 0.1	0.034	1.29 ± 0.3	1.18 ± 0.1	0.05

Supplementary Table 2: Median concentrations of 130 metabolites, 7 calculated summary variables and 13 calculated ratio variables measured using the TMIC PRIME® Assay in 122 adult SAM survivors and 90 age, sex and BMI-matched community participants.

		Metabolites	Community Participants	SAM Survivors	SAM Survivors Vs Community Participants				Oedematous Malnutrition	Severe Wasting	Oedematous Malnutrition Vs Severe Wasting			
			n = 90	n = 122	β	SE	*p*	FDR -*p*	n = 53	n = 69	β	SE	*p*	FDR -*p*
**Biogenic Amine, µM**	1	Creatinine	69 [58.6-84.3]	73.5 [61.1-86.7]	-4.4	2.23	0.049	0.082	76 [60-85.8]	73 [62-86.8]	-1.77	2.67	0.51	0.97
	2	Taurine	93.1 [66.2-115]	115 [82.6-132]	-2.05	0.487	<0.001	<0.001	116 [82.4-131]	114 [83.4-132]	-0.62	0.683	0.37	0.97
	3	Putrescine	0.071 [0.042-0.090]	0.063 [0.047-0.087]	-0.00522	0.0139	0.71	0.76	0.07 [0.04-0.09]	0.0627 [0.05-0.0903]	0.00702	0.0184	0.70	0.97
	4	Trans-Hydroxyproline	15.4 [11.1-24.9]	16.5 [13.2-24.1]	-0.032	0.0227	0.16	0.23	16.1 [13-23.1]	17.7 [13.5-24.1]	0.0253	0.0301	0.40	0.97
	5	Alpha-Aminoadipic acid	0.35 [0.22-0.50]	0.32 [0.24-0.41]	0.0483	0.0567	0.40	0.46	0.30 [0.24-0.37]	0.33 [0.25-0.43]	0.0751	0.0666	0.26	0.97
	6	Methionine sulfoxide	0.55 [0.42-0.71]	0.67 [0.56-0.72]	-0.107	0.0343	0.0020	0.0055	0.66 [0.55-0.71]	0.67 [0.59-0.73]	0.0285	0.04	0.48	0.97
	7	Serotonin	0.48 [0.37-0.69]	0.61 [0.47-0.84]	-0.205	0.0661	0.0022	0.0058	0.64 [0.47-0.83]	0.60 [0.46-0.88]	-0.0312	0.0928	0.74	0.97
	8	Asymmetric Dimethylarginine	0.40 [0.32-0.46]	0.37 [0.31-0.43]	0.014	0.0185	0.45	0.51	0.365 [0.314-0.412]	0.375 [0.313-0.441]	0.0416	0.023	0.074	0.97
	9	Total Dimethylarginine	1.95 [1.13-2.56]	1.52 [1.23-1.85]	0.123	0.0511	0.017	0.032	1.43 [1.18-1.83]	1.56 [1.36-1.85]	0.0527	0.0559	0.35	0.97
	10	Kynurenine	1.58 [1.23-1.98]	1.72 [1.38-2.06]	-0.159	0.0664	0.018	0.033	1.67 [1.35-2]	1.79 [1.39-2.27]	0.139	0.0793	0.083	0.97
	11	Spermidine	0.138 [0.0982-0.176]	0.164 [0.126-0.224]	-0.085	0.0206	<0.001	<0.001	0.155 [0.117-0.229]	0.171 [0.135-0.222]	0.0244	0.0286	0.40	0.97
	12	Spermine	0.151 [0.145-0.162]	0.165 [0.156-0.171]	-1.99	0.54	<0.001	0.00101	0.166 [0.155-0.169]	0.164 [0.158-0.171]	0.571	0.707	0.42	0.97
	13	Sarcosine	1 [0.8-1.23]	0.843 [0.609-1.18]	0.162	0.0543	0.0032	0.0078	0.792 [0.597-1.1]	0.857 [0.621-1.21]	0.0549	0.0851	0.52	0.97
**Amino Acid, µM**	1	Glycine	299 [243-346]	311 [258-362]	-2.81	1.99	0.16	0.23	311 [258-342]	311 [261-368]	1.4	2.52	0.58	0.97
	2	Alanine	294 [252-338]	288 [248-323]	1.43	8.69	0.87	0.89	285 [247-323]	288 [248-325]	0.838	9.37	0.93	0.98
	3	Serine	121 [108-135]	126 [112-141]	-7.35	3.34	0.029	0.051	118 [110-135]	129 [114-143]	6.82	4.07	0.097	0.97
	4	Proline	154 [120-188]	153 [130-184]	-0.00594	0.0101	0.56	0.62	153 [132-188]	154 [130-184]	-0.00104	0.0119	0.93	0.98
	5	Valine	172 [149-190]	187 [172-209]	-301	62.8	<0.001	<0.001	194 [171-215]	186 [173-207]	-45.7	85.6	0.60	0.97
	6	Threonine	97 [77.8-118]	120 [97.2-135]	-5.49	1.12	<0.001	<0.001	109 [89.6-133]	125 [106-135]	1.77	1.52	0.25	0.97
	7	Leucine	122 [107-136]	136 [120-157]	-50.4	9.48	<0.001	<0.001	135 [119-160]	136 [123-156]	-7.02	12.4	0.57	0.97
	8	Isoleucine	57.3 [51.6-64.4]	61.2 [55-67]	-68.4	25.3	0.0074	0.016	60.3 [53.7-67.3]	61.6 [57.3-66.8]	12	29.3	0.68	0.97
	9	Asparagine	41.4 [36.8-47.9]	44 [37.6-48.2]	-2.18	1.15	0.060	0.096	43.1 [36-47.9]	44.3 [39-49.1]	-0.0651	1.32	0.96	0.98
	10	Aspartic acid	19.6 [16.4-26.6]	30.3 [22.5-41.1]	-0.101	0.0179	<0.001	<0.001	29.7 [22.4-37.3]	30.6 [24.2-42.3]	0.025	0.0264	0.35	0.97
	11	Glutamine	584 [528-655]	516 [462-575]	194	48.4	<0.001	<0.001	502 [456-569]	526 [481-578]	29.6	50.4	0.56	0.97
	12	Glutamic acid	61.2 [50-75.5]	74.8 [62.2-88.6]	-0.222	0.045	<0.001	<0.001	77.5 [65.9-89.4]	69.6 [60.8-88.5]	-0.0326	0.0623	0.60	0.97

	13	Methionine	23 [19.4-26.6]	24.8 [21.8-29.1]	-8.59	2.26	<0.001	<0.001	23.7 [21-26.9]	25.4 [23.2-29.8]	3.36	2.66	0.21	0.97
	14	Histidine	72.8 [67.1-79.8]	75.5 [69.6-82.1]	-274	112	0.016	0.030	75.5 [68.6-81.8]	75.5 [71-82.8]	27.9	146	0.85	0.98
	15	Phenylalanine	64.7 [58-71.1]	68.6 [62.2-75.3]	-89.2	28.9	0.0023	0.0058	68.5 [62.3-74.7]	68.9 [62.1-77.6]	26.7	34.9	0.45	0.97
	16	Arginine	112 [95.6-138]	114 [99.4-123]	0.52	3.72	0.89	0.90	114 [96-122]	113 [102-129]	4.96	4.32	0.25	0.97
	17	Citrulline	23.4 [19.8-29.2]	25 [20.9-29.5]	-1.5	0.861	0.083	0.13	24.7 [20-28.5]	25.1 [21.1-30.3]	0.341	1.03	0.74	0.97
	18	Tyrosine	55.6 [49.5-64.2]	52.4 [45.5-63.6]	1.96	1.9	0.30	0.38	52.3 [44.6-63.1]	52.4 [45.6-63.8]	2.2	2.21	0.32	0.97
	19	Tryptophan	48.4 [42.3-53.6]	41.7 [36.5-46.3]	10.3	2.7	<0.001	<0.001	41.7 [35.7-45.4]	41.7 [37-47]	0.643	3	0.83	0.97
	20	Ornithine	56.4 [41.9-66.6]	61.4 [49.7-74.9]	-0.464	0.138	<0.001	0.00276	61.5 [50.1-74]	61.3 [49.6-75.9]	-0.0861	0.179	0.63	0.97
	21	Lysine	148 [130-186]	152 [136-176]	0.206	1.11	0.85	0.88	149 [131-182]	156 [139-171]	1.4	1.27	0.27	0.97
	22	Betaine	35.4 [28.8-44.8]	42.7 [33.1-51]	-0.508	0.126	<0.001	<0.001	40.5 [31.9-48]	43.9 [33.6-52]	0.0507	0.17	0.77	0.97
**Lysophophatidylcholine, µM**	1	LysoPC a C14:0	3.22 [2.76-3.85]	3.74 [2.8-4.73]	-0.0975	0.0515	0.060	0.096	4.08 [2.7-5.01]	3.59 [2.85-4.45]	-0.0614	0.0743	0.41	0.97
	2	LysoPC a C16:1	1.62 [1.35-2.06]	1.98 [1.59-2.46]	-0.144	0.0334	<0.001	<0.001	1.95 [1.62-2.45]	2.02 [1.57-2.46]	0.0179	0.0473	0.71	0.97
	3	LysoPC a C16:0	70.9 [61.4-84.3]	86.3 [72.7-104]	-0.403	0.0775	<0.001	<0.001	85.2 [73.1-105]	87.6 [72.6-100]	-0.0433	0.113	0.70	0.97
	4	LysoPC a C17:0	0.869 [0.706-1.13]	1.13 [0.881-1.35]	-0.182	0.0447	<0.001	<0.001	1.11 [0.853-1.33]	1.18 [0.936-1.36]	0.0468	0.0618	0.45	0.97
	5	LysoPC a C18:2	22.1 [17-25.6]	24.2 [18.5-29.4]	-0.121	0.0409	0.0036	0.0087	23.8 [17.9-29.8]	24.5 [19.1-29]	-0.046	0.0589	0.44	0.97
	6	LysoPC a C18:1	12.7 [10-15.4]	14.5 [11.5-17.5]	-0.143	0.0379	<0.001	<0.001	14.4 [11-16.8]	14.7 [11.6-17.8]	-0.0124	0.0525	0.81	0.97
	7	LysoPC a C18:0	21.7 [17.8-26]	26.3 [20.5-29.8]	-0.717	0.17	<0.001	<0.001	27.1 [20.7-31.2]	24.6 [20.2-29]	-0.292	0.256	0.26	0.97
	8	LysoPC a C20:4	5.18 [4.24-6.24]	5.94 [5.15-7.29]	-0.154	0.04	<0.001	<0.001	6.22 [5.19-7.46]	5.88 [4.96-7.1]	-0.0799	0.0544	0.15	0.97
	9	LysoPC a C20:3	3.63 [3.19-3.93]	2.92 [2.52-3.45]	0.52	0.0863	<0.001	<0.001	3.06 [2.56-3.56]	2.85 [2.5-3.38]	-0.18	0.131	0.17	0.97
	10	LysoPC a C24:0	0.2 [0.107-0.298]	0.224 [0.152-0.262]	-0.00869	0.0299	0.77	0.81	0.232 [0.142-0.271]	0.221 [0.169-0.253]	-0.015	0.0369	0.68	0.97
	11	LysoPC a C26:1	0.0938 [0.0674-0.116]	0.107 [0.0788-0.133]	-0.0477	0.0173	0.0062	0.014	0.106 [0.0821-0.134]	0.11 [0.0786-0.132]	-0.00385	0.0228	0.87	0.98
	12	LysoPC a C26:0	0.649 [0.515-0.857]	0.8 [0.632-1.04]	-0.167	0.0459	<0.001	0.0012	0.776 [0.617-0.946]	0.837 [0.636-1.05]	0.112	0.0616	0.071	0.97
	13	LysoPC a C28:1	0.291 [0.209-0.37]	0.334 [0.274-0.406]	-0.0616	0.0246	0.013	0.026	0.333 [0.273-0.403]	0.335 [0.276-0.407]	0.0142	0.0305	0.64	0.97
	14	LysoPC a C28:0	0.364 [0.302-0.435]	0.416 [0.335-0.489]	-0.0653	0.0256	0.012	0.023	0.423 [0.333-0.515]	0.415 [0.343-0.484]	-0.0109	0.0328	0.74	0.97
**Sphingomyelin, µM**	1	SM (OH) C14:1	3.15 [2.46-3.66]	3.1 [2.65-3.74]	-0.0423	0.0423	0.32	0.39	3.19 [2.58-3.74]	3.05 [2.72-3.74]	0.068	0.0603	0.26	0.97
	2	SM (OH) C16:1	1.87 [1.66-2.28]	1.96 [1.66-2.35]	-0.0335	0.034	0.33	0.39	1.92 [1.66-2.35]	1.97 [1.67-2.35]	0.0491	0.0468	0.30	0.97
	3	SM (OH) C22:1	10.5 [9.15-12.6]	8.45 [7.25-10.6]	0.219	0.0336	<0.001	<0.001	8.59 [7.35-10.8]	8.16 [7.12-10.4]	-0.0164	0.051	0.75	0.97
	4	SM (OH) C22:2	8.73 [7.3-10.5]	7.49 [6.09-8.96]	0.279	0.0458	<0.001	<0.001	7.7 [6.21-9.26]	7.41 [6.06-8.43]	-0.00404	0.0688	0.95	0.98
	5	SM (OH) C24:1	1.13 [0.977-1.34]	1.45 [1.17-1.71]	-0.226	0.04	<0.001	<0.001	1.48 [1.25-1.71]	1.44 [1.12-1.71]	0.009	0.0555	0.87	0.98
	6	SM C16:0	93.7 [82.5-103]	94.4 [84.2-109]	-0.133	0.103	0.2	0.27	94.1 [84.2-110]	95.7 [85.2-108]	0.0476	0.145	0.74	0.97
	7	SM C16:1	11.8 [10.1-13.5]	12.2 [10.3-14.5]	-0.0277	0.0275	0.32	0.39	12.6 [10.7-14.8]	11.8 [9.85-14.2]	0.0103	0.0394	0.79	0.97

	8	SM C18:1	6.81 [5.46-9.06]	7.51 [5.65-9.44]	-0.0195	0.0427	0.65	0.71	7.49 [5.78-10]	7.53 [5.5-9.03]	0.0366	0.0566	0.52	0.97
	9	SM C18:0	12.2 [9.93-14.2]	13.3 [10.5-15.9]	-0.175	0.0686	0.011	0.023	13.8 [11.2-15.1]	12.9 [10.2-16]	0.0249	0.0983	0.80	0.97
	10	SM C20:2	0.453 [0.361-0.588]	0.467 [0.384-0.571]	-0.0124	0.0294	0.67	0.73	0.476 [0.425-0.576]	0.438 [0.35-0.563]	-0.0159	0.0409	0.70	0.97
**Phosphatidylcholine, μM**	1	PC acyl-alkyl (ae) C36:0	0.515 [0.457-0.611]	0.768 [0.565-0.947]	-0.346	0.0427	<0.001	<0.001	0.77 [0.581-0.97]	0.747 [0.534-0.936]	0.00495	0.0695	0.94	0.98
	2	PC acyl-alkyl (ae) C40:6	2.12 [1.71-2.41]	2.35 [1.89-2.95]	-0.123	0.04	0.0023	0.0058	2.37 [1.89-2.85]	2.28 [1.92-3.01]	0.0517	0.0606	0.40	0.97
	3	PC diacyl (aa) C36:6	0.267 [0.204-0.342]	0.374 [0.267-0.482]	-0.304	0.0547	<0.001	<0.001	0.403 [0.275-0.496]	0.325 [0.266-0.482]	-0.0103	0.0801	0.90	0.98
	4	PC diacyl (aa) C36:0	2.27 [2.05-2.9]	2.87 [2.27-3.44]	-0.129	0.0257	<0.001	<0.001	2.91 [2.48-3.38]	2.76 [2.21-3.54]	0.00188	0.0384	0.96	0.98
	5	PC diacyl (aa) C32:2	3.8 [2.97-4.91]	3.87 [2.84-4.89]	0.0574	0.0444	0.20	0.27	3.9 [3.06-5.16]	3.59 [2.6-4.57]	-0.00854	0.0604	0.89	0.98
	6	PC diacyl (aa) C38:6	38.8 [28.8-47.4]	43.9 [35.3-57.5]	-0.167	0.0411	<0.001	<0.001	43.7 [36.1-56.1]	43.9 [35.2-58.2]	0.0405	0.0619	0.52	0.97
	7	PC diacyl (aa) C38:0	1.82 [1.44-2.18]	2.14 [1.73-2.65]	-0.17	0.045	<0.001	<0.001	2.23 [1.74-2.63]	2.02 [1.7-2.85]	0.0314	0.0681	0.65	0.97
	8	PC diacyl (aa) C40:6	14.8 [11.3-20.3]	18.6 [13.7-24.5]	-0.299	0.0901	0.0011	0.0030	19.2 [14.2-25.5]	17.9 [13.6-24.5]	0.00804	0.135	0.95	0.98
	9	PC diacyl (aa) C40:2	0.546 [0.457-0.639]	0.521 [0.467-0.61]	0.01	0.0311	0.75	0.79	0.518 [0.468-0.609]	0.528 [0.467-0.611]	-0.0146	0.0433	0.74	0.97
	10	PC diacyl (aa) C40:1	0.421 [0.342-0.469]	0.435 [0.361-0.514]	-0.0711	0.0336	0.036	0.061	0.442 [0.365-0.525]	0.427 [0.36-0.495]	-0.0303	0.0487	0.54	0.97
**Acylcarnitine, μM**	1	C0	32.4 [27.2-36.3]	31.3 [27.2-36.2]	0.0468	0.0593	0.43	0.49	32.1 [27.4-36.8]	31.2 [27.2-35.5]	0.0408	0.0859	0.64	0.97
	2	C2	7.94 [5.91-9.54]	6.55 [5.05-8.21]	0.271	0.0761	<0.001	0.00144	6.52 [5.19-8.23]	6.57 [5.01-8.14]	0.0632	0.107	0.56	0.97
	3	C3:1	0.0336 [0.0155-0.0392]	0.0166 [0.0136-0.0214]	1.76	0.307	<0.001	<0.001	0.0152 [0.0132-0.0211]	0.0175 [0.0145-0.0215]	0.161	0.358	0.65	0.97
	4	C3	0.217 [0.181-0.287]	0.196 [0.17-0.25]	0.15	0.0637	0.020	0.036	0.192 [0.172-0.244]	0.198 [0.169-0.266]	0.0464	0.0882	0.60	0.97
	5	C4:1	0.0259 [0.015-0.0358]	0.0164 [0.014-0.0202]	0.911	0.19	<0.001	<0.001	0.0159 [0.014-0.02]	0.0167 [0.0143-0.0211]	0.00251	0.227	0.99	0.99
	6	C4	0.141 [0.114-0.193]	0.134 [0.106-0.166]	0.216	0.108	0.047	0.078	0.126 [0.104-0.166]	0.14 [0.11-0.166]	0.108	0.154	0.48	0.97
	7	C3-OH	0.0276 [0.0225-0.0327]	0.0217 [0.0182-0.0261]	0.0868	0.02	<0.001	<0.001	0.0228 [0.0189-0.027]	0.0214 [0.0179-0.0257]	-0.0295	0.0259	0.26	0.97
	8	C5:1	0.021 [0.018-0.0264]	0.0189 [0.0156-0.0234]	0.103	0.0439	0.020	0.036	0.0182 [0.0157-0.0208]	0.0192 [0.0153-0.0245]	0.0651	0.0603	0.28	0.97
	9	C5	0.105 [0.0853-0.137]	0.108 [0.0819-0.149]	-0.00636	0.117	0.96	0.96	0.115 [0.0839-0.157]	0.1 [0.0797-0.147]	-0.0245	0.159	0.88	0.98
	10	C4-OH	0.0591 [0.0451-0.0745]	0.0652 [0.0453-0.0923]	-0.115	0.0731	0.12	0.18	0.0674 [0.0423-0.0925]	0.0609 [0.0484-0.0916]	-0.0621	0.106	0.56	0.97
	11	C6:1 (Hexenoylcarnitine)	0.0276 [0.0242-0.0322]	0.0246 [0.0212-0.0288]	0.129	0.0377	<0.001	0.0022	0.0242 [0.0211-0.0281]	0.0249 [0.0215-0.0295]	0.00514	0.0543	0.93	0.98
	12	C6 (C4:1-DC) (Hexanoylcarnitine)	0.0747 [0.0582-0.0936]	0.0619 [0.0508-0.0816]	0.146	0.056	0.0096	0.020	0.0629 [0.0498-0.0832]	0.0609 [0.0523-0.0767]	-0.0414	0.0734	0.57	0.97
	13	C5-OH (C3-DC-M) (hydroxyvalerylcarnitine)	0.0232 [0.0196-0.0294]	0.0274 [0.0222-0.0331]	-0.427	0.124	<0.001	0.0021	0.029 [0.0215-0.0352]	0.0255 [0.0224-0.031]	-0.1	0.156	0.52	0.97
	14	C5:1-DC (Glutaconylcarnitine)	0.0188 [0.0158-0.0222]	0.0149 [0.0126-0.0177]	0.245	0.0383	<0.001	<0.001	0.0144 [0.0124-0.0176]	0.0151 [0.013-0.018]	0.0483	0.0523	0.36	0.97
	15	C5-DC (C6-OH)(Glutarylcarnitine)	0.0176 [0.016-0.0212]	0.0168 [0.0136-0.0202]	0.00994	0.00875	0.26	0.33	0.0171 [0.0132-0.0216]	0.0163 [0.014-0.0195]	-0.00165	0.0119	0.89	0.98
	16	C8 (Octanoylcarnitine)	0.155 [0.107-0.221]	0.105 [0.079-0.155]	0.292	0.0715	<0.001	<0.001	0.104 [0.0741-0.155]	0.108 [0.0825-0.155]	-0.0218	0.101	0.83	0.97
	17	C5-M-DC (methylglutarylcarnitine)	0.0195 [0.0161-0.0222]	0.0212 [0.0174-0.0252]	-0.0196	0.009	0.031	0.054	0.0207 [0.0175-0.0248]	0.0218 [0.0172-0.0257]	0.00281	0.0124	0.82	0.97
	18	C9 (Nonaylcarnitine)	0.0214 [0.0187-0.0278]	0.0186 [0.0159-0.0247]	0.115	0.0479	0.017	0.032	0.0183 [0.0167-0.0247]	0.019 [0.0155-0.0247]	-0.0801	0.0622	0.20	0.97

	19	C7-DC (pimelylcarnitine)	0.0728 [0.0538-0.125]	0.13 [0.0744-0.27]	-1.18	0.308	<0.001	<0.001	0.125 [0.0765-0.297]	0.135 [0.0697-0.238]	-0.433	0.442	0.33	0.97
	20	C10:2 (decadienylcarnitine)	0.0325 [0.026-0.0435]	0.0328 [0.0252-0.039]	0.0485	0.0487	0.32	0.39	0.0339 [0.0266-0.039]	0.032 [0.0249-0.0389]	-0.0521	0.0602	0.39	0.97
	21	C10:1 (Decenoylcarnitine)	0.25 [0.185-0.318]	0.217 [0.186-0.295]	0.0763	0.0525	0.15	0.22	0.239 [0.18-0.279]	0.213 [0.187-0.302]	-0.0178	0.0667	0.79	0.97
	22	C10 (Decanoylcarnitine)	0.268 [0.18-0.39]	0.188 [0.142-0.283]	0.287	0.0706	<0.001	<0.001	0.188 [0.135-0.279]	0.189 [0.149-0.284]	-0.0234	0.0945	0.81	0.97
	23	C12:1 (Dodecenoylcarnitine)	0.184 [0.136-0.262]	0.211 [0.161-0.267]	-0.0787	0.0621	0.21	0.27	0.205 [0.159-0.247]	0.223 [0.169-0.279]	0.0267	0.088	0.76	0.97
	24	C12 (dodecanoylcarnitine)	0.0948 [0.078-0.12]	0.079 [0.0646-0.107]	0.143	0.0503	0.0049	0.012	0.0772 [0.0619-0.107]	0.0794 [0.0708-0.106]	0.000899	0.0704	0.99	0.99
	25	C14:2 (Tetradecadienylcarnitine)	0.0882 [0.0646-0.128]	0.074 [0.0534-0.108]	0.149	0.0725	0.041	0.069	0.0745 [0.0555-0.102]	0.0726 [0.0525-0.112]	-0.0372	0.102	0.72	0.97
	26	C14:1 (tetradecenoyl carnitine)	0.184 [0.144-0.235]	0.173 [0.136-0.211]	0.0686	0.049	0.16	0.23	0.171 [0.138-0.208]	0.174 [0.133-0.212]	-0.00245	0.0669	0.97	0.98
	27	C14 (tetradecanoylcarnitine)	0.0377 [0.0294-0.0509]	0.0293 [0.0223-0.0372]	0.275	0.0573	<0.001	<0.001	0.0294 [0.023-0.0358]	0.0292 [0.0221-0.0377]	-0.0199	0.0727	0.79	0.97
	28	C12-DC (dodecanedioylcarnitine)	0.0152 [0.012-0.0188]	0.0156 [0.0133-0.0182]	-0.000312	0.000661	0.64	0.70	0.0163 [0.0141-0.019]	0.0148 [0.0128-0.0178]	-0.00137	0.000804	0.091	0.97
	29	C14:2-OH (hydroxytetradecadienylcarnitine)	0.0138 [0.011-0.0171]	0.0116 [0.00902-0.0155]	0.0098	0.00385	0.012	0.023	0.0114 [0.0087-0.0144]	0.0125 [0.0091-0.016]	0.00231	0.00534	0.67	0.97
	30	C14:1-OH (Hydroxytetradecenoyl carnitine)	0.0176 [0.014-0.0231]	0.0158 [0.0124-0.0221]	0.0227	0.0164	0.17	0.24	0.0147 [0.0119-0.0202]	0.0168 [0.0135-0.0231]	0.0188	0.0214	0.38	0.97
	31	C16:2 (Hexadecadienylcarnitine)	0.0172 [0.0131-0.0238]	0.0146 [0.0116-0.0211]	0.0746	0.0687	0.28	0.35	0.0169 [0.0126-0.0223]	0.014 [0.0114-0.0193]	-0.118	0.0907	0.20	0.97
	32	C16:1 (Hexadecenoylcarnitine)	0.0577 [0.0486-0.0665]	0.0614 [0.051-0.0737]	-0.035	0.0223	0.12	0.18	0.0657 [0.0549-0.0763]	0.058 [0.0493-0.071]	-0.0306	0.0329	0.36	0.97
	33	C16 (Hexadecanoylcarnitine)	0.136 [0.109-0.161]	0.124 [0.106-0.143]	0.084	0.0379	0.028	0.049	0.121 [0.101-0.141]	0.126 [0.108-0.147]	0.0395	0.0492	0.42	0.97
	34	C16:2-OH (hydroxyhexadecadienylcarnitine)	0.0112 [0.00848-0.0137]	0.0098 [0.00765-0.0123]	0.00931	0.0034	0.0068	0.015	0.0098 [0.0074-0.0118]	0.0098 [0.0078-0.0125]	0.00174	0.0042	0.68	0.97
	35	C16:1-OH (Hydroxyhexadecenoylcarnitine)	0.0134 [0.0114-0.0174]	0.0148 [0.0112-0.0182]	-0.00932	0.00977	0.34	0.40	0.0152 [0.0117-0.0182]	0.0148 [0.0112-0.0181]	0.00664	0.0143	0.64	0.97
	36	C16-OH (hydroxyhexadecanoylcarnitine)	0.0097 [0.00762-0.0141]	0.00965 [0.008-0.0136]	-0.0136	0.057	0.81	0.84	0.0095 [0.008-0.0119]	0.01 [0.008-0.0151]	0.0302	0.0746	0.69	0.97
	37	C18:2 (Octadecadienylcarnitine)	0.109 [0.0901-0.129]	0.104 [0.0822-0.129]	0.0634	0.0409	0.12	0.18	0.106 [0.0871-0.131]	0.103 [0.0776-0.119]	-0.102	0.0563	0.071	0.97
	38	C18:1 (Octadecenoylcarnitine)	0.167 [0.124-0.202]	0.153 [0.128-0.184]	0.0206	0.0212	0.33	0.40	0.151 [0.128-0.173]	0.159 [0.128-0.194]	0.0146	0.0268	0.59	0.97
	39	C18 (Octadecanoylcarnitine)	0.046 [0.035-0.0589]	0.0393 [0.0313-0.048]	0.481	0.146	0.0011	0.0031	0.0393 [0.0293-0.0473]	0.0395 [0.0322-0.0481]	0.0879	0.188	0.64	0.97
	40	C18:1-OH (Hydroxyoctadecenoylcarnitine)	0.0134 [0.0106-0.0184]	0.0159 [0.0104-0.02]	-0.0141	0.015	0.35	0.41	0.0158 [0.0105-0.0186]	0.0159 [0.0104-0.0205]	0.0162	0.0216	0.46	0.97
**Organic Acid, μM**	1	Lactic acid	1960 [1600-2350]	1940 [1610-2330]	-0.0138	0.0409	0.74	0.78	2010 [1650-2300]	1870 [1600-2340]	-0.0667	0.0563	0.24	0.97
	2	Beta-Hydroxybutyric acid	98.9 [40.7-187]	76.9 [26.2-157]	0.392	0.142	0.0063	0.014	76.7 [29.3-143]	77.1 [26.2-163]	0.181	0.193	0.35	0.97
	3	Alpha-Ketoglutaric acid	5.8 [4.82-6.85]	6.07 [5.32-7.24]	-0.0801	0.0225	<0.001	0.0014	6.43 [5.46-7.32]	5.93 [5.29-6.94]	-0.0189	0.0304	0.54	0.97
	4	Citric acid	98.5 [81.9-112]	94 [80.8-107]	0.0437	0.0302	0.15	0.22	92.4 [77-106]	96 [82-107]	0.028	0.0407	0.49	0.97
	5	Butyric acid	0.588 [0.418-0.962]	0.952 [0.468-1.26]	-0.19	0.0727	0.0096	0.020	0.874 [0.368-1.23]	0.997 [0.518-1.33]	0.0329	0.11	0.77	0.97
	6	3-(3-Hydroxyphenyl)-3-hydroxypropanoic acid (HPHPA)	0.0427 [0.0242-0.0856]	0.0436 [0.0227-0.118]	-0.0745	0.132	0.57	0.64	0.0495 [0.0214-0.121]	0.0434 [0.0248-0.114]	-0.158	0.189	0.41	0.97
	7	P-Hydroxyhippuric acid	0.176 [0.158-0.222]	0.154 [0.143-0.185]	0.0508	0.0186	0.0069	0.015	0.153 [0.143-0.177]	0.155 [0.144-0.191]	0.026	0.0213	0.22	0.97
	8	Succinic acid	3.71 [3.02-4.06]	2.94 [2.49-3.73]	0.117	0.039	0.0031	0.0077	2.86 [2.49-3.7]	3.07 [2.5-3.74]	0.0205	0.0581	0.72	0.97
	9	Fumaric acid	0.659 [0.553-0.809]	0.602 [0.465-0.767]	0.0618	0.0376	0.10	0.16	0.613 [0.484-0.766]	0.596 [0.462-0.767]	-0.082	0.059	0.17	0.97

	10	Pyruvic acid	55.9 [45-70.1]	48.2 [37.8-55.3]	1.82	0.483	<0.001	<0.001	48.2 [39.9-54.9]	48.3 [37.7-55.3]	-0.129	0.575	0.82	0.97
	11	Isobutyric acid	0.818 [0.625-1.24]	1.19 [0.696-1.39]	-0.0863	0.0644	0.18	0.25	1.19 [0.58-1.4]	1.19 [0.762-1.38]	0.0181	0.0928	0.85	0.98
	12	Hippuric acid	1.1 [0.686-1.78]	1.25 [0.761-1.9]	-0.178	0.131	0.18	0.25	1.23 [0.747-1.96]	1.27 [0.783-1.89]	-0.0254	0.168	0.88	0.98
	13	Methylmalonic acid	0.0731 [0.0505-0.0996]	0.0748 [0.0566-0.11]	-0.365	0.329	0.27	0.34	0.0773 [0.0612-0.111]	0.0712 [0.0509-0.101]	-0.513	0.432	0.24	0.97
	14	Homovanillic acid	0.0574 [0.0472-0.0741]	0.0542 [0.0406-0.08]	0.0726	0.0592	0.22	0.29	0.0573 [0.0435-0.0846]	0.0518 [0.0394-0.0731]	-0.179	0.0918	0.053	0.97
	15	Indole acetic acid	1.08 [0.705-1.6]	1.2 [0.843-1.65]	-0.069	0.078	0.38	0.44	1.13 [0.771-1.89]	1.21 [0.906-1.56]	-0.0332	0.1	0.74	0.97
	16	Uric acid	305 [254-363]	289 [244-348]	8.13	10.2	0.43	0.49	302 [250-353]	281 [242-339]	-10	13.1	0.44	0.97
**Monosaccharide, µM**	1	Glucose	4240 [3920-4580]	4110 [3820-4460]	0.000125	0.000118	0.29	0.37	4100 [3760-4370]	4130 [3930-4470]	0.000198	0.00015	0.19	0.97
**Other, µM**	1	Methylhistidine	6.02 [4.35-8.94]	7.22 [5.06-10.2]	-0.138	0.0707	0.052	0.085	7.7 [4.27-10.3]	6.77 [5.44-9.67]	0.0202	0.0904	0.82	0.97
	2	Creatine	17.3 [12.1-27.4]	19.8 [14.2-26.3]	-0.0818	0.0611	0.18	0.25	19.2 [15.3-25.7]	20 [13.9-27]	0.178	0.0776	0.024	0.97
	3	Choline	11.2 [9.14-13.3]	13.2 [11.1-15.8]	-0.643	0.132	<0.001	<0.001	13.2 [11.2-14.9]	13 [11.1-15.9]	0.0601	0.168	0.72	0.97
	4	Trimethylamine N-oxide (TMAO)	1.99 [1.37-2.47]	1.94 [1.36-2.55]	-0.0392	0.0864	0.65	0.71	1.96 [1.39-2.58]	1.89 [1.35-2.34]	-0.0517	0.107	0.63	0.97
**Summary Variable, µM**	1	Total Amino Acids	2600 [2450-2820]	2640 [2440-2800]	-118000	122000	0.33	0.40	2630 [2430-2780]	2650 [2490-2820]	162000	124000	0.20	0.97
	2	Essential amino acids	829 [734-904]	869 [812-955]	-55700	15000	<0.001	<0.001	862 [772-950]	872 [820-955]	6570	18600	0.73	0.97
	3	Aromatic amino acids (AAAs)	241 [227-270]	239 [221-264]	-62.9	1130	0.96	0.96	236 [218-264]	240 [222-264]	1070	1260	0.40	0.97
	4	Glucogenic amino acids	1990 [1870-2180]	1980 [1850-2130]	-21300	74300	0.78	0.81	1970 [1830-2120]	1980 [1860-2130]	101000	80300	0.21	0.97
	5	Ketogenic amino acids	274 [243-324]	290 [264-317]	-151	72.4	0.039	0.066	293 [251-332]	290 [273-313]	30.9	84.3	0.72	0.97
	6	Branched chain amino acids (BCAAs)	348 [311-388]	386 [351-432]	-1560	315	<0.001	<0.001	390 [347-439]	384 [357-425]	-194	418	0.64	0.97
	7	Urea cycle derivatives	224 [193-253]	239 [212-267]	-22.8	6.15	<0.001	<0.001	236 [212-267]	245 [212-271]	2	8.05	0.80	0.97
**Ratio**	1	Branched chain : Aromatic amino acids (BCAA/AAA)	1.44 [1.32-1.54]	1.65 [1.42-1.8]	-0.119	0.0197	<0.001	<0.001	1.66 [1.53-1.81]	1.63 [1.39-1.79]	-0.025	0.0298	0.40	0.97
	2	Fischer Ratio	2.92 [2.62-3.1]	3.26 [2.75-3.58]	-0.181	0.0375	<0.001	<0.001	3.27 [2.9-3.62]	3.26 [2.73-3.55]	-0.0675	0.0561	0.23	0.97
	3	Kynurenine : Tryptophan	0.0327 [0.0266-0.0424]	0.0412 [0.0343-0.0519]	-0.12	0.0242	<0.001	<0.001	0.0413 [0.0343-0.052]	0.041 [0.0343-0.0517]	0.0446	0.0305	0.15	0.97
	4	Ornithine : Arginine	0.465 [0.398-0.572]	0.53 [0.432-0.668]	-0.124	0.0476	0.010	0.021	0.552 [0.452-0.659]	0.52 [0.425-0.669]	-0.0752	0.0689	0.28	0.97
	5	Putrescine : Ornithine	844 [555-1310]	949 [700-1330]	-0.00661	0.00519	0.20	0.27	933 [723-1500]	955 [692-1300]	-0.00361	0.00682	0.60	0.97
	6	Serotonin : Tryptophan	0.0109 [0.0084-0.0153]	0.0141 [0.0109-0.0217]	-0.293	0.0695	<0.001	<0.001	0.0141 [0.0109-0.0225]	0.0142 [0.0106-0.021]	-0.035	0.0975	0.72	0.97
	7	Spermidine : Putrescine	2.1 [1.41-3.3]	2.82 [1.95-3.65]	-0.246	0.0871	0.0053	0.012	2.75 [1.85-3.94]	2.88 [2.01-3.53]	0.013	0.121	0.92	0.98
	8	Spermine : Spermidine	1.17 [0.876-1.59]	0.975 [0.761-1.31]	0.196	0.0562	<0.001	0.0019	1.03 [0.775-1.36]	0.908 [0.759-1.27]	-0.0544	0.0766	0.48	0.97
	9	Tyrosine : Phenylalanine	0.852 [0.732-1.03]	0.755 [0.663-0.892]	0.12	0.034	<0.001	0.0016	0.762 [0.653-0.895]	0.734 [0.666-0.882]	0.0248	0.0466	0.60	0.97
	10	Total dimethylarginine : L-Arginine	0.0166 [0.0109-0.0228]	0.0132 [0.0104-0.0173]	0.141	0.0551	0.011	0.023	0.0123 [0.01-0.0167]	0.0136 [0.0111-0.0173]	0.00468	0.0662	0.94	0.98
	11	Citrulline : Ornithine	0.424 [0.352-0.512]	0.4 [0.328-0.486]	0.0479	0.037	0.20	0.27	0.398 [0.322-0.466]	0.405 [0.337-0.497]	0.0409	0.0481	0.40	0.97
	12	Citrulline : Arginine	0.202 [0.164-0.265]	0.218 [0.181-0.267]	-0.055	0.0296	0.064	0.10	0.216 [0.196-0.252]	0.219 [0.177-0.272]	-0.0237	0.0413	0.57	0.97

	13	Acetylcarnitine : Free carnitine (C2:C0)	0.243 [0.185-0.309]	0.213 [0.154-0.276]	0.12	0.0358	<0.001	0.0028	0.213 [0.16-0.278]	0.218 [0.152-0.26]	0.0174	0.0473	0.71	0.97

Supplementary Table 3: Models examining the relationship between β-hydroxybutyric acid and acylcarnitine-to-carnitine ratio with L/S ratio or mean liver attenuation in SAM survivors and community participants

	**β-Hydroxybutyric acid**			adj. R^2 0.21	**Acylcarnitine to free carnitine ratio (C2/C0)**			adj. R^2 0.33
	Est.	SE	*p*	Part. R^2	Est.	SE	*p*	Part. R^2
**Mean liver attenuation**	-7.6	2.1	< 0.001	0.076	-0.0062	0.0014	< 0.0001	0.11
SAM vs. Control	60	19	0.0018	0.060	0.040	0.013	0.0021	0.058
Age	-2.3	1.2	0.060	0.018	-0.0015	0.0008	0.064	0.017
Sex	43	20	0.037	0.023	0.062	0.014	< 0.0001	0.11
BMI	1.8	2.2	0.42	0	0.0023	0.0015	0.13	0.009

	**β-Hydroxybutyric acid**			adj. R^2 0.21	**Acylcarnitine to free carnitine ratio (C2/C0)**			adj. R^2 0.40
	Est.	SE	*p*	Part. R^2	Est.	SE	*p*	Part. R^2
**L/S ratio**	-213	81	0.0093	0.050	-0.19	0.053	< 0.001	0.094
SAM vs. Control	52	20	0.0094	0.049	0.040	0.013	0.0027	0.068
Age	-2.5	1.2	0.047	0.026	-0.002	0.001	0.011	0.047
Sex	49	20	0.017	0.040	0.071	0.013	< 0.0001	0.19
BMI	3.4	2.4	0.16	0.0086	0.003	0.002	0.032	0.031

Supplementary Table 4: Variables included in SNF analyses as clinical and body composition variables

	Clinical variables		Body composition variables	
1	Age, years		1	Bone Mineral Density, g/cm^2
2	SAM exposure (Yes / no)		2	Tissue Fat, g
3	SAM type (severe wasting / edematous / none)		3	Total mass, kg
4	Sex (male / female)		4	Fat, g
5	Education level		5	Lean, g
		0=None	6	Bone mineral content, g
		1=Infant/kindergarten/basic	7	Android Fat, g
		2=Primary/all age	8	Gynoid Fat, g
		3=New secondary	9	Android-to-Gynoid fat ratio
		4=Secondary high	10	Trunk-to-total fat ratio
		5=Some college	11	Legs-to-total fat ratio
		6=College graduate	12	Arms/legs-to-trunk fat ratio
		7=Some university	13	Android tissue, g
		8=University graduate	14	Android fat, g
6	Smoke now (yes / no)		15	Android lean mass, g
7	Number of cigarettes per day		16	Android bone min content, g
8	Years smoking		17	Android total, kg
9	Drank alcohol 12 times in the past year? (yes/ no)		18	Arms tissue, g
10	Marital status		19	Arms fat tissue, g
		Married /common law-yes	20	Arms lean, g
		Widowed/ divorced/ separated- yes	21	Arms bone mineral content, g
		Single-yes	22	Gynoid tissue, g
		Visiting relationship-yes	23	Gynoid fat, g
11	Occupation		24	Gynoid lean tissue, g
		1-Unemployed	25	Gynoid bone mineral content, g
		2-Employed	26	Legs tissue, g
		3-Self-employed	27	Legs lean mass, g
		4-Home maker	28	Legs bone mineral content, g
		5-Student	29	Trunk tissue, g

		6-Retired	30	Trunk lean mass, g
		1-Unemployed	31	Trunk bone mineral content, g
12	Paid work (yes / no)		32	Bone mineral density spine
13	Fasting glucose		33	z-score spine
14	Fasting insulin		34	Bone mineral density femur
15	HOMA-IR		35	z-score femur
16	Mean systolic blood pressure, mmHg			
17	Mean diastolic blood pressure, mmHg			
18	Height, cm			
19	Weight, kg			
20	BMI			
21	Waist Circumference, cm			

Excluded variables due to missingness: SES indices (n=35, 17%), WBISI (n=81, 38%), IGI (n=79, 37%), oDI (n=81, 38%), mean liver attenuation (n=61, 29%), L/S ratio, (n=90, 42%)
